# Tonsil explants as a human *in vitro* model to study vaccine responses

**DOI:** 10.3389/fimmu.2024.1425455

**Published:** 2024-09-17

**Authors:** Elena Bonaiti, Manuele G. Muraro, Philippe A. Robert, Jens Jakscha, Stefan Dirnhofer, Ivan Martin, Christoph T. Berger

**Affiliations:** ^1^ Translational Immunology, Department of Biomedicine, University of Basel, Basel, Switzerland; ^2^ Tissue Engineering, Department of Biomedicine, University of Basel and University Hospital Basel, Basel, Switzerland; ^3^ Ear Nose Throat Clinic, University Hospital Basel, Basel, Switzerland; ^4^ Institute of Medical Genetics and Pathology, University Hospital Basel, Basel, Switzerland; ^5^ Department of Biomedical Engineering, University of Basel, Allschwil, Switzerland; ^6^ University Center of Immunology, University Hospital Basel, Basel, Switzerland

**Keywords:** tonsil, bioreactor, *in vitro* model, vaccines, influenza, antibodies, human

## Abstract

**Introduction:**

Vaccination is one of the most effective infection prevention strategies. Viruses with high mutation rates -such as influenza- escape vaccine-induced immunity and represent significant challenges to vaccine design. Influenza vaccine strain selection is based on circulating strains and immunogenicity testing in animal models with limited predictive outcomes for vaccine effectiveness in humans.

**Methods:**

We developed a human *in vitro* vaccination model using human tonsil tissue explants cultured in 3D perfusion bioreactors to be utilized as a platform to test and improve vaccines.

**Results:**

Tonsils cultured in bioreactors showed higher viability, metabolic activity, and more robust immune responses than those in static cultures. The *in vitro* vaccination system responded to various premanufactured vaccines, protein antigens, and antigen combinations. In particular, a multivalent *in vitro* immunization with three phylogenetically distant H3N2 influenza strains showed evidence for broader B cell activation and induced higher antibody cross-reactivity than combinations with more related strains. Moreover, we demonstrate the capacity of our *in vitro* model to generate de novo humoral immune responses to a model antigen.

**Discussion:**

Perfusion-cultured tonsil tissue may be a valuable human *in vitro* model for immunology research with potential application in vaccine candidate selection.

## Introduction

The development of vaccines dramatically reduced the burden of infectious diseases. Vaccination stimulates the immune response against the specific pathogen to establish an immunological memory and, thereby, long-lasting protection from disease (1). Vaccines can also induce heterologous off-target effects, including protection against non-related pathogens, by the enhanced response of trained innate immune cells (2, 3). Over the past two decades, vaccines have prevented an estimated 50 million deaths, and projections indicate that by 2030, they will have averted 97 million deaths (4). Despite these remarkable achievements, a need remains to develop or improve vaccines, especially against viruses with high mutation rates like HIV and influenza, to induce broadly neutralizing antibodies covering mutated viruses (5, 6).

The effectiveness of a vaccine depends on various factors, including the individual pre-existing immune history, age, or the combination of vaccine antigens and adjuvants used (1, 7, 8). A successful experimental vaccine model takes these factors into account and has the capacity to capture the critical features of the human immune response. However, most preclinical vaccine studies are still performed in animals, not in human *in vitro* models. Murine models inform on the global biology of immune responses and their dynamics in a standardized, genetically identical model. Notably, these models can only recapitulate shared global features between the murine and human immune systems, and it is unclear how well these similarities translate to humans. Discrepancies between the human and murine immune systems have been described in B and T cell signaling pathways, Ig isotypes, cytokine receptors, costimulatory molecule expression, and function (9–11). Furthermore, human-specific infectious agents may need adaptation to infect or replicate in non-human hosts. In influenza, for example, the virus adaptation requires repeated passages in mouse lungs that result in viral adaptations by introducing amino acid changes, allowing its replication in mice *in vivo.* Moreover, mice must be infected with high influenza virus titers (12–14). Additionally, various animal models have been employed depending on the pathogen (e.g., ferrets for influenza), making it challenging to identify which predicted vaccine responses are specific to a model or transferable to humans (13, 15, 16).

Non-human primate models (NHP) represent the setting closest to humans and are valid options to recapitulate human infections such as HIV, tuberculosis, or influenza for pre-clinical studies (17–19). However, NHPs are more challenging to maintain and expensive compared to mice, and the number of animals and vaccine scenarios necessary to reproduce human features like preexisting immunity or patient heterogeneity is ethically not at reach using NHPs. Therefore, intensive efforts are needed to develop human *in vitro* systems that accurately reflect critical properties of human immune responses and can be used for high-throughput prediction of vaccine efficacy.

Human organoids and engineered tissue approaches are emerging as promising tools to overcome inter-species differences for more accurate vaccine testing. Different strategies have been developed to recapitulate salient features of secondary lymphoid organs and drive B cell responses (20). Engineered *in vitro* models need to include multiple heterogeneous cell populations derived from humans or mice, seeded into scaffolds that allow the spatial organization and interactions of cells to mimic a tissue (21). Giese et al. combined different subsets from peripheral blood mononuclear cells (PBMC) with scaffolds in bioreactors to monitor the immune response upon antigen exposure (22, 23), while others developed B cell follicle organoids, allowing the differentiation of murine naive B cells into antigen-specific germinal center (GC) B cells (24–26). Most recently, the potential of human tonsil single-cells for developing *in vitro* systems that resemble lymphoid organs and support adaptive immunity, including germinal center reactions, has been demonstrated (27–29). Compared to single-cell suspension models, tissue explants may more closely reflect the physiologic 3D spatial organization and cell-cell interactions found in *in vivo* tissues. In particular, single-cell suspensions from tonsils may lack the stromal network support that acts synergically with the immune cells in generating the adaptive immune response. Moreover, the formation of chemical gradients in culture plates and batch-fed bioreactors may affect cell aggregation and, thereby, the immune response. Therefore, *in vitro* systems have the potential to mimic human immunity, but it is essential to assess the capacity of different model designs to reproduce key features of the human vaccine response to reach predictive systems for application in vaccine development.

Here, we used human tonsil explants to develop an *ex vivo* lymphoid tissue culture system to investigate vaccine responses *in vitro*. We defined the capacity of a 3D perfusion system to preserve the cellular organ architecture and to recapitulate immune activation in different antigen and adjuvant conditions. We then focused on the *in vitro* response to influenza antigens. Influenza was chosen as a model due to the variability of circulating influenza strains and the vast presence of preexisting immunity in humans, which is challenging to study in mice but present in tonsil immune cells (30, 31). Influenza viruses frequently accumulate mutations in the hemagglutinin (HA) surface glycoprotein, the main target of antibodies, necessitating yearly strain updates to vaccines. Thus, we used our *in vitro* model to assess how immunizations with different combinations of influenza strains could affect the breadth of the antibody response. Altogether, we provide an *in vitro* model of human vaccine response that recapitulates numerous important complex features of human immunity to different vaccine regimens, including *de novo* B cell responses.

## Materials and methods

### Tissue preparation and culture conditions

Human palatine tonsils were obtained from adult patients (19-45 years old) undergoing tonsillectomy at the Ear Nose Throat Clinic. The ethics committee of Northwestern Switzerland approved the study, and patients gave written informed consent. For sample preparation, we first removed macroscopically identified necrotic and fibrotic areas from the tonsils. The vital-appearing tissue was dissected into approximately 3 mm cubic pieces.

The U-CUP bioreactor (Cellec Biotek AG) was used to culture tonsil samples under perfusion, following the procedure outlined in patent WO2015181185A1. This method has previously been demonstrated to reliably maintain tissue architecture and microenvironment in *ex vivo* tumor tissue cultures (32–34). In brief, three tonsil pieces were sandwiched between two discs of type I collagen (Ultrafoam collagen haemostat from Davol, Inc.) and assembled in the perfusion chamber according to the manufacturer’s instructions. The bioreactors were then filled with 8 mL of RPMI medium supplemented with 10% heat-inactivated fetal bovine serum (FBS), 1x Glutamax, 1x nonessential amino acids, and 1x penicillin-streptomycin (all Gibco). The bioreactors were connected to a syringe pump system (Programmable Harvard Apparatus PHD ULTRA 2000) and set to maintain a bidirectional flow rate of 0.47mL/min (including a 1-minute pause between flow reversals to reduce shear stress), which shows good tissue perfusion (35). In parallel, tonsil tissue pieces were cultured in a static petri dish culture using the same media (8 mL) as a control. All cell cultures were conducted at 37°C in a 5% CO2 humidified atmosphere. The experimental setup is shown in **Supplementary Figure S1**.

We included various antigens and stimuli for *in vitro* immunization or stimulation as indicated in the respective experiments. We used a commercial inactivated quadrivalent influenza split-vaccine (VaxigripTetra^®^, Sanofi Pasteur) and a 13-valent pneumococcal conjugate vaccine (PCV13 (Prevenar-13^®^), Pfizer) as well as TLR agonists (TLR4, lipopolysaccharide (LPS) (10 ng/mL; from Escherichia coli O111:B4; Sigma-Aldrich, St. Louis, MO, USA), and TLR9, CpG (0,4 ug/mL; Human ODN 2006; InvivoGen, San Diego, CA, USA). To test for *de novo* antigen responses, we used recombinant Keyhole Limpet Hemocyanin (KLH) (100 ng/mL; Calbiochem^®^) and for antigen combination stimulation experiments recombinant HA proteins (H1N1 A/California/07/2009, H3N2 A/Victoria/361/2011, H3N2 A/Kansas/14/2017, H3N2 A/Cambodia/e0826360/2020, H3N2 A/Hong Kong/2671/2019, H3N2 A/Singapore/INFIMH-16-0019/2016, H3N2 A/Darwin/9/2021 (100 ng/mL; all eEnzyme) and H3N2 A/Hong Kong/1/1968 (100 ng/mL; Sino Biological).

### Sampling and tissue processing

Tissue culture supernatants were sampled at the defined time points indicated in the respective experiments, centrifuged, and stored at -80°C until measurements. At the end of the tissue culture, the tonsil pieces were split for histopathological and cellular analysis. For multiparameter flow cytometric and metabolic analyses, single-cell suspensions were generated by Collagenase/Dispase (Roche^®^, Switzerland) digestion (continuous shaking at 37°C for 1h) followed by passage through a 70-µm cell strainer (Corning^®^) and washing twice in PBS. The tissue piece was fixed in 4% paraformaldehyde for 24h at 4°C and then paraffin-embedded for histology.

### Blood sample preparation

Peripheral blood samples from healthy blood donors and, for a subset, tonsil donor-matched peripheral blood samples were obtained. The serum was collected and stored at -80°C before use for antibody detection. PBMC were isolated by density gradient using Lymphoprep™ (Axis-Shield) and used immediately. For functional experiments, PBMC were seeded at 1x10^6^ cells/200 µL in 96-well plates in complete RPMI medium and stimulated with the same conditions as the tonsil pieces.

### Flow cytometry-based immunophenotyping

We performed multiparameter flow cytometry for immunophenotyping and viability assessment. Cells were incubated for 20 min at 4°C with Fixable Viability Dye eFluor™ 780 (eBioscience™) in PBS. Next, cells were stained in FACS buffer (PBS + 2% FBS) for 20 min at 4°C with the following anti-human antibodies in different panels (‘lymphocyte subsets’, ‘B cell differentiation’, ‘T cell differentiation’, and ‘T cell activation’): CD3 (SK7) Alexa Fluor^®^ 700, CD4 (OKT4) BV421, CD8 (SK1) Alexa Fluor^®^ 488, CD14 (63D3) FITC, CD19 (HIB19) PE-Cy7, CD19 (HIB19) BV421, CD27 (O323) PE, CD38 (HIT2) FITC, CD45RA (HI100) BV605, CD62L (DREG-56) PE-Cy7, HLA-DR (L243) PE-Cy7 (all Biolegend^®^). Samples were acquired on BD LSRFortessa™ flow cytometer, and data were analyzed using FlowJo v10.7.1 software (Tree Star). We gated on lymphocytes to exclude stromal cells based on size and granularity. The gating strategies are displayed in **Supplementary Figure S2** and **Supplementary Figure S4**.

### Cytokine measurements

We measured the levels of TNF-α, IL-13, IL-4, IL-10, IL-6, IL-2, TNF-β, INFγ, IL-17A, IL-12p70, APRIL, BAFF, CD40L in the supernatants of perfusion-cultured tonsils using the LEGENDplex™ Human B Cell Panel (Biolegend^®^), following the manufacturer’s instruction. Data were acquired on BD LSRFortessa™ flow cytometer and then analyzed using LEGENDplex™ Data Analysis Software Suite (Biolegend^®^).

### Cell metabolic activity

Cellular fitness of perfusion-cultured tonsils was determined using the MTT Assay Kit (Abcam), following the manufacturer’s instructions. We used 1x106 cells per condition of a tissue single-cell suspension. The absorbance values of the samples were measured at 590 nm using the SynergyH1 spectrophotometer (BioTek) connected to the program Gen5. Results were normalized to the values obtained for the corresponding tonsil cells at day 0 and, as such, expressed as ‘% cellular fitness’.

### Antibody detection

Antibody production in the cell culture was measured by ELISA or an influenza-strain-specific multiplex assay. To detect total IgG, ELISA plates (Thermo Fisher Scientific) were coated with the anti-human IgG capture monoclonal antibody at 2.5 µg/mL (Mabtech). Undiluted culture supernatants were added to the wells and incubated for 2 h. After washing, anti-human IgG detection antibodies diluted to 0.5 µg/mL (Mabtech) and streptavidin-horseradish peroxidase (HRP) enzyme (Peprotech) were added to detect the total IgG antibodies. Plates were developed using TMB Substrate Solution (Thermo Fisher Scientific), the reactions were stopped with 50 µL of Stop Solution (Thermo Fisher Scientific), and the absorbances were determined at 450 nm using the SynergyH1 spectrophotometer (BioTek).

Anti-pneumococcus IgG was detected in the supernatants using the Human Anti-Streptococcus pneumoniae IgG ELISA Kit (Abbexa^®^), according to the manufacturer’s instructions.

To detect influenza-specific antibodies, the HA of different influenza A strains were coupled to different MagPlex^®^ microspheres (Luminex Corporation), following the manufacturer’s instruction. In particular, 2 µg of each type of HA protein was used to couple one million microspheres. To measure the HA-specific antibodies, 20 µL of cell culture supernatant or serum was added to 20 µL of microsphere mixture (containing 50 microspheres/µL of each type) and then incubated for 1 h at room temperature. After two washes with PBS containing Tween-20, the microspheres were incubated with PE mouse anti-human IgG (SouthernBiotech), -IgA (Miltenyi Biotec), or -IgM (Biolegend^®^) for 30 min at room temperature. The influenza-specific antibodies were quantified using a Luminex^®^ 100 machine running on xPonent software (Luminex Corporation).

### Histology and immunohistology

Paraffin-embedded tissues were sectioned at 5 µm on a microtome and mounted on Superfrost Plus slides. Sections were dried overnight at 42°C and then stained with haematoxylin and eosin (H&E) using a standard protocol by Gemini autostainer. Immunohistochemistry (IHC) staining was done using the Ventana Discovery Ultra autostainer with the following anti-human antibodies: CD3 (2GV6, cat# 790-4341, Ventana), CD20 (L26, cat# 760-2531, Ventana), CD21 (EP3093, cat# 760-4438, Ventana), and Ki67 (Mib-1, cat# IR626, Dako). Images were acquired with a Nikon Ti2 microscope (Nikon) using x20 magnification, exported as TIFF files, and analyzed using QuPath software, version 0.3.2 (36). Cell density was determined by automated counting of the cell number per total area of each image field. The frequency of CD3, CD20, and CD21 positive cells was calculated as the percentage of DAB-positive area versus total area. Proliferation was quantified by counting the number of Ki67-positive cells versus the total area.

### Statistical analyses

Data are expressed as mean +/- standard deviation (SD). Group comparisons were performed using a two-way analysis of variance (ANOVA) followed by Turkey’s or Bonferroni’s *post-hoc* tests as appropriate. Otherwise, the Mann-Whitney test for single comparison and the Friedman test with Dunn’s correction for multiple comparisons were used. A p-value of less than 0.05 was considered significant. All the statistical analyses were implemented in GraphPad Prism 9 software (GraphPad).

## Results

### Distinct composition and distribution of immune cells in the tonsils and peripheral blood compartment

**Figure 1:**
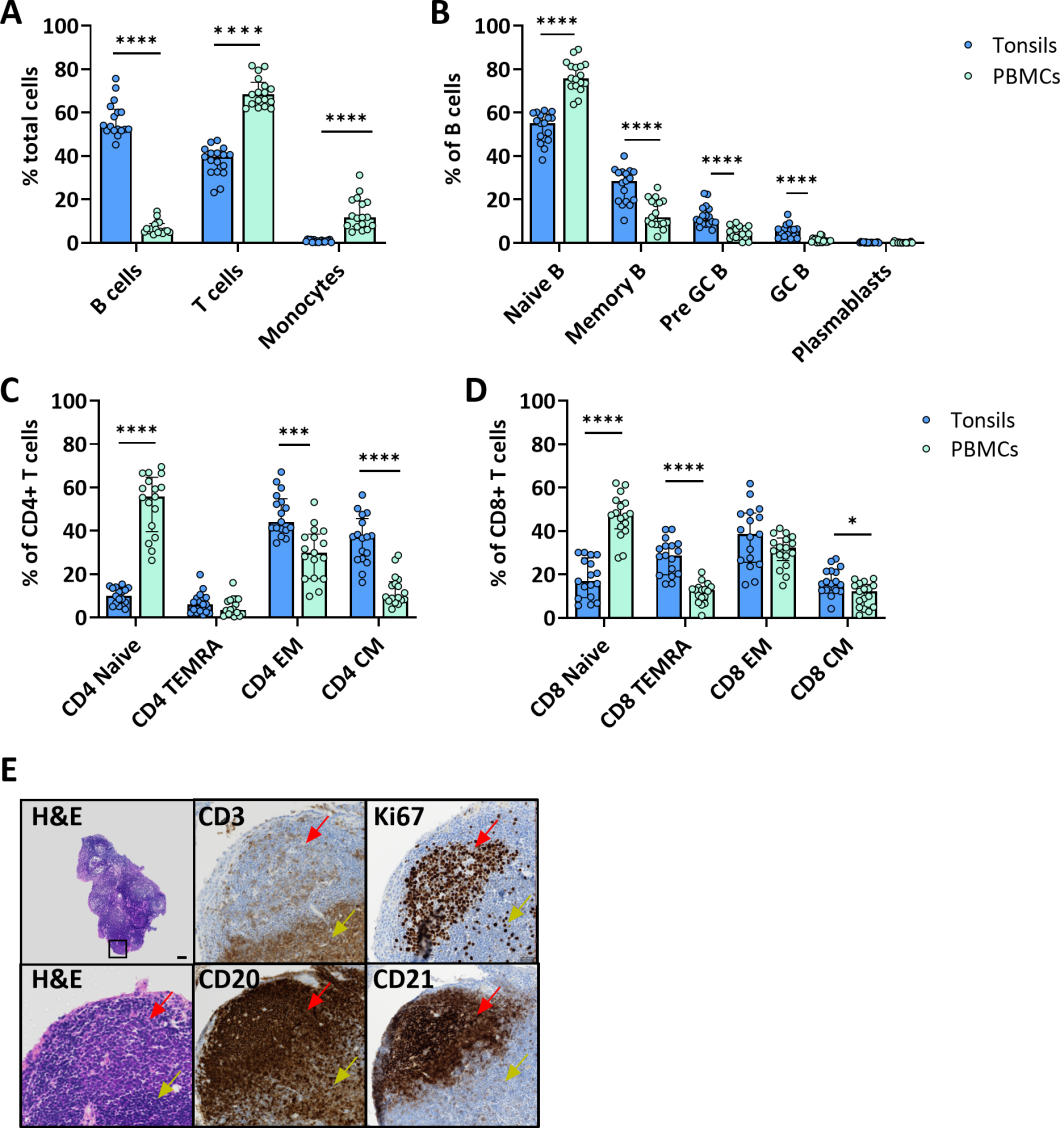
Tonsil-derived and peripheral blood immune cells have a distinct immunophenotype. **(A)** Cell composition of immune cell types in freshly isolated tonsil cells and PBMC from blood donors. Frequencies were determined by flow cytometry (n=17). Frequencies of the main B cell **(B)** and CD4+ **(C)** and CD8+ **(D)** T cell differentiation states in tonsil vs PBMC determined by flow cytometry (n=17). **(E)** Representative H&E and immunohistochemistry for CD3, CD20, CD21 and Ki67 of *ex vivo* tonsils showing the architecture of the secondary lymphoid organ. In the top left H&E image, the black square indicates the region that is magnified in the other panels. Red arrows indicate the B cell follicles and yellow arrows the T cell area. Scale bar = 50 µm. Values were compared with two-way ANOVA with Bonferroni’s *post hoc* test. *p<0.005, ***p<0.0001, ****p<0.0001.

We assessed the immune cell composition of *ex vivo* tonsils compared to the immunophenotype in peripheral blood mononuclear cells (PBMC) by multiparameter flow cytometry. Overall, B cells were significantly more frequent in tonsils than in PBMC (56.9 vs. 6.9%, p<0.0001, respectively) and showed phenotypic differences (**Figure 1A**). Specifically, memory (26.5 vs. 13.7% CD27^+^ CD38^-^), pre-germinal center (pre-GC) (12.4 vs. 4.8% CD27^-^ CD38^+^), and GC (5.5 vs. 1.2% CD27^+^ CD38^+^) B cells were more abundant in the lymphoid tissue (**Figure 1B**). Conversely, PBMC contained significantly larger fractions of T cells (37.6 vs. 69.3%, p<0.0001) and monocytes (0.9 vs. 13.4%, p<0.0001) (**Figure 1A**). However, amongst T cells, tonsils harbored more memory CD4^+^ and CD8^+^ T cells, especially effector memory (EM), central memory (CM) and terminally differentiated effector memory (TEMRA) (**Figures 1C, D**).

Next, we investigated the spatial distribution of different immune cell types in tonsil explants by immunohistochemistry for T cells (CD3), B cells (CD20), and FDCs (CD21) markers, as well as the proliferation marker Ki67. B cells and FDCs were predominantly found in the follicles surrounded by T cell-rich areas. The proliferation rate was higher in cells within the lymphoid follicles (**Figure 1E**).

### 3D perfusion bioreactor increases *ex vivo* tonsil viability

Next, we assessed if tonsil explants maintain their structural and functional characteristics during extended *in vitro* culture. We compared using a 3D perfusion bioreactor, which features a closed system and continuous flow of culture medium, to a static culture condition, where tonsil pieces are kept in tissue culture dishes. Antigen-specific (quadrivalent inactivated influenza vaccine; QIV) and non-specific (CpG) B cell stimuli were used to assess the functional capacity of the cultured tissues for immune activation.

**Figure 2:**
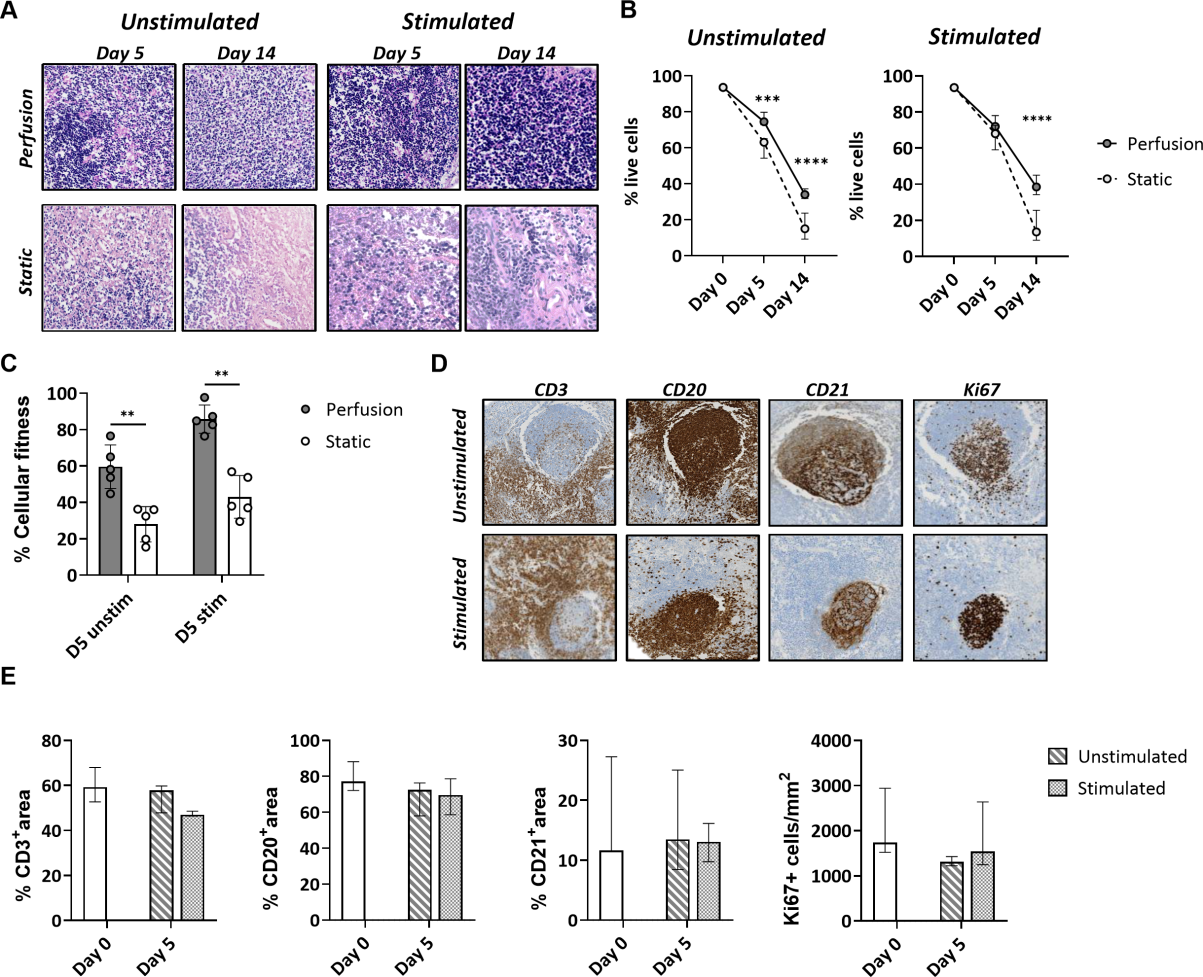
Cell survival and cellular fitness of tonsils in the 3D perfusion bioreactor vs static system. **(A)** Representative H&E images of tonsil tissues cultured in 3D perfusion bioreactor vs static system for 5 and 14 days. **(B)** Cell survival quantified by flow cytometry at day 0, day 5, and day 14 in unstimulated and quadrivalent influenza vaccine (QIV)-stimulated conditions (n=7). Plotted values are the mean +/- SD. **(C)** Metabolic cell activity (‘cellular fitness’) in an MTT assay at day 5 in unstimulated and QIV-stimulated conditions (n=5). Similar results were obtained with CpG stimulations (not shown). **(D)** Maintenance of the lymphoid organ structure was assessed in perfusion-cultured tonsils stained for the markers CD3, CD20, CD21, and Ki67 at day 5. **(E)** The CD3/CD20/CD21 positive area and the frequency of Ki67^+^ cells were assessed by automated image analysis (n=3). Statistical significance between groups was determined using two-way ANOVA with Bonferroni’s *post hoc* test. **p<0.001, ***p<0.0001, ****p<0.0001.

After 5 and 14 days of culture, tonsils in 3D perfusion bioreactors maintained a higher cell density compared to those in the static system (**Figure 2A**; **Supplementary Figure S3**). Quantification of the viability of tonsil cells by flow cytometry indicated higher viability over time (75.9 ± 3.5% at day 5 and 35.9 ± 7.1% at day 14 for the unstimulated condition) in tonsils cultured in bioreactors. Contrarily, cells cultured in the static system exhibited a marked decline in viability, especially at day 14 (61.1 ± 5.4 at day 5 and 15.5 ± 7.3% at day 14 for the unstimulated condition) (**Figure 2B**). Next, we employed an MTT assay that assesses the cellular metabolic activity in the two culture systems by measuring NAD(P)H-dependent oxidoreductase enzyme activity. We observed a significant difference in cellular fitness between tonsils cultured in the bioreactor vs. the static system already at day 5 (relative cellular fitness 59.6 ± 11.9% vs. 28.0 ± 9.7% for the unstimulated condition; 85.8 ± 7.8% vs. 43.0 ± 11.7% after stimulation) (**Figure 2C**).

Complementing the viability analysis, we quantified the percentage of T cell, B cell, and FDC areas versus total tissue area for the tonsils cultured in the bioreactor. Expression of the markers CD3, CD20, and CD21 was preserved in the presence or absence of antigen at day 5 (**Figure 2D**). Proliferating Ki67+ cells were mainly localized in the lymphoid follicles, especially in tonsils stimulated with antigen (**Figure 2D**).

Taken together, the data showed that the 3D perfusion bioreactor provides a higher cell viability and metabolic activity of tonsils compared to static cultures and preserves the original morphology and immunological architecture.

### 3D perfusion culture allows the generation of robust *in vitro* B cell responses

**Figure 3:**
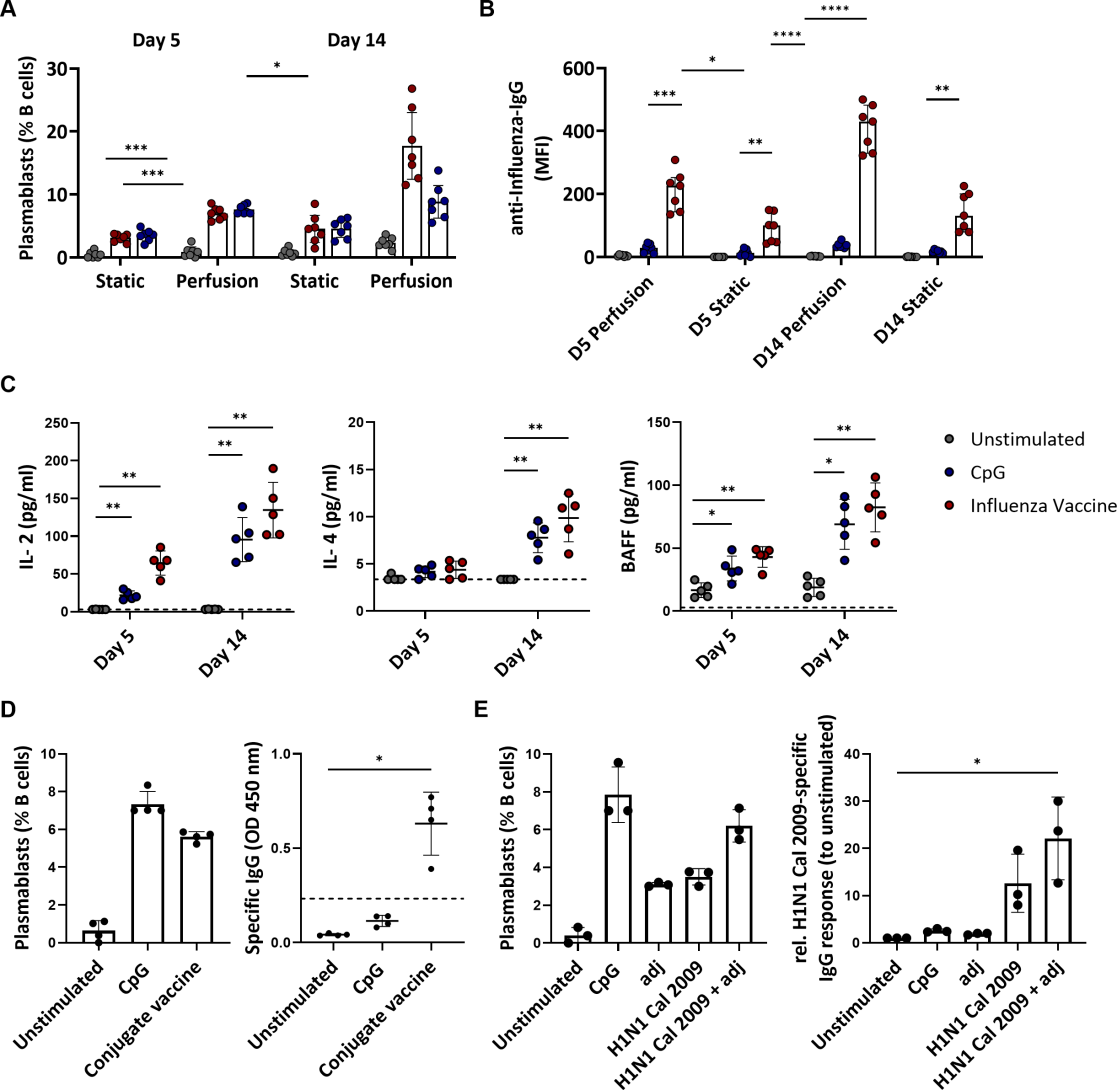
Adaptive immune responses to vaccines and single-protein antigens in tonsils cultured in 3D perfusion bioreactor vs static system. **(A)** Plasmablast differentiation in unstimulated, CpG- and influenza vaccine-stimulated tonsils (n=7) at days 5 and 14. Flow cytometry-based immunophenotyping. **(B)** Influenza-specific IgG antibodies (Luminex) in supernatants of tissue cultures (n=7) using bioreactors vs static system at days 5 and 14 in the indicated conditions. **(C)** Cytokine concentrations in the culture supernatants in response to CpG and influenza vaccine stimulation in tonsil tissues (n=5) cultured in 3D perfusion bioreactors. **(D)** Plasmablast differentiation and pneumococcal specific-IgG production in response to the pneumococcal conjugate vaccine PCV13 (n=4). The dotted line indicates the cut-off value for test positivity of the pneumococcal-specific IgG. **(E)** Plasmablast differentiation and H1N1 Cal 2009-specific IgG response (normalized to unstimulated condition) upon 5-day stimulation in perfusion-cultured tonsils (n=3). Two-way ANOVA with Turkey’s *post hoc* test and Friedman test with Dunn’s correction were used to determine the significance values in the graphs a-c and d-e, respectively. BAFF= B cell activating factor; adj = adjuvant (=LPS). *p<0.005, **p<0.001, ***p<0.0001, ****p<0.0001.

The end-points of a physiological B cell response to antigen stimulation are the production of plasmablasts and memory cells. A wave of circulating plasmablasts becomes detectable in the blood of immunized human subjects a few days post-immunization (37). Therefore, we assessed the plasmablast level by flow cytometry as a surrogate for the cellular immune response of the cultured tonsils. Stimulation with the influenza vaccine or BCR-independent stimulation with CpG increased the frequency of plasmablasts compared to the unstimulated conditions. This increase was significantly higher in tonsils cultured in the 3D perfusion bioreactor than in the static system (CpG: 7.59 vs. 3.49%, p=0.0009 at day 5 and 8.83 vs. 4.57%, p=0.0858 at day 14; influenza vaccine: 6.99 vs. 3.11%, p=0.0010 at day 5 and 17.72 vs. 4.54%, p=0.0154 at day 14) (**Figure 3A**). The magnitude of plasmablast differentiation associated with antibody production: influenza-specific IgG antibodies increased upon influenza vaccine stimulation, especially in the supernatant of perfusion-cultured tonsils (MFI 410 vs. 143 at day 14 in bioreactor vs static system) (**Figure 3B**). Total IgG did not change upon influenza vaccine stimulation (37.8 μg/mL at day 5, 25.9 μg/mL at day 14 in the bioreactor) but increased with CpG (251.4 μg/mL, 443.8 μg/mL) compatible with a non-specific polyclonal B cell stimulation (**Supplementary Figure S5**). In addition, we measured the levels of 13 cytokines involved in B cell functions and B cell activation, proliferation, and survival (TNF-α, IL-13, IL-4, IL-10, IL-6, IL-2, TNF-β, INFγ, IL-17A, IL-12p70, APRIL, BAFF, CD40L) in the supernatant of perfusion-cultured tonsils. We found that the development of the humoral responses in the bioreactors was associated with increased levels of IL-2, IL-4, and the B cell activation factor BAFF compared to the unstimulated condition (**Figure 3C**). Moreover, stimulation with the influenza vaccine or CpG resulted in increased IL-6 levels in the culture supernatants, suggesting innate cell activation as well as IFNγ and TNF which may indicate Th1 helper responses (**Supplementary Figure S6**). Our data, therefore, support that perfusion-cultured tonsils respond to immune stimulation and are suitable for comparing the immune responses to vaccine stimulation. Thus, we focused on the 3D perfusion bioreactor for the subsequent experiments.

Next, we investigated whether perfusion-cultured tonsils could support a specific B cell response to polysaccharide-conjugate vaccines. Since the stimulation with the influenza vaccine indicated immunological activity upon stimulation already at day 5, with higher viability than at the later time point (day 14), we focused on day 5. Using the commercial pneumococcal conjugate vaccine (PCV13), we observed an increase in plasmablasts (0.64 vs. 5.61% for the unstimulated vs. PCV13 condition) and robust production of anti-pneumococcal IgG (OD 0.04 vs. 0.63, p=0.0140) (**Figure 3D**). This supports that our *in vitro* system allows testing the humoral response to various types of premanufactured vaccines.

Next, we investigated whether the perfusion-cultured tonsils could serve as a platform for analyzing vaccine antigen composition. First, we stimulated the tonsil tissues with a single protein antigen in the presence or absence of an adjuvant. Specifically, we used the HA from H1N1 California/07/2009, for which we expected a high seroprevalence due to the long time this strain has been circulating in humans, and a TLR4 agonist as adjuvant. The recombinant HA of H1N1 Cal 2009 in combination with the adjuvant tends to induce a higher level of plasmablasts (6.2 vs. 3.5%) and HA-specific IgG compared to stimulation with the recombinant protein alone (1.75-fold difference in H1-specific IgG levels normalized to unstimulated condition) (**Figure 3E**). Therefore, the antigen alone induced an *in vitro* B cell response which was higher in the presence of the adjuvants achieving levels of plasmablast induction comparable to that of polyclonal CpG stimulation.

Altogether, these data show that the 3D perfusion culture system better supports the generation of an adaptive immune response -i.e., plasmablast differentiation, antibody production, and B cell stimulating cytokine profiles- than a static culture system. Furthermore, perfusion-cultured tonsils may be suitable to study B cell responses to different types of vaccines and to test different antigens, antigen combinations, and adjuvants.

### Multivalent influenza strain *in vitro* immunization of perfusion-cultured tonsils induces a broader humoral response compared to monovalent stimulations

**Figure 4:**
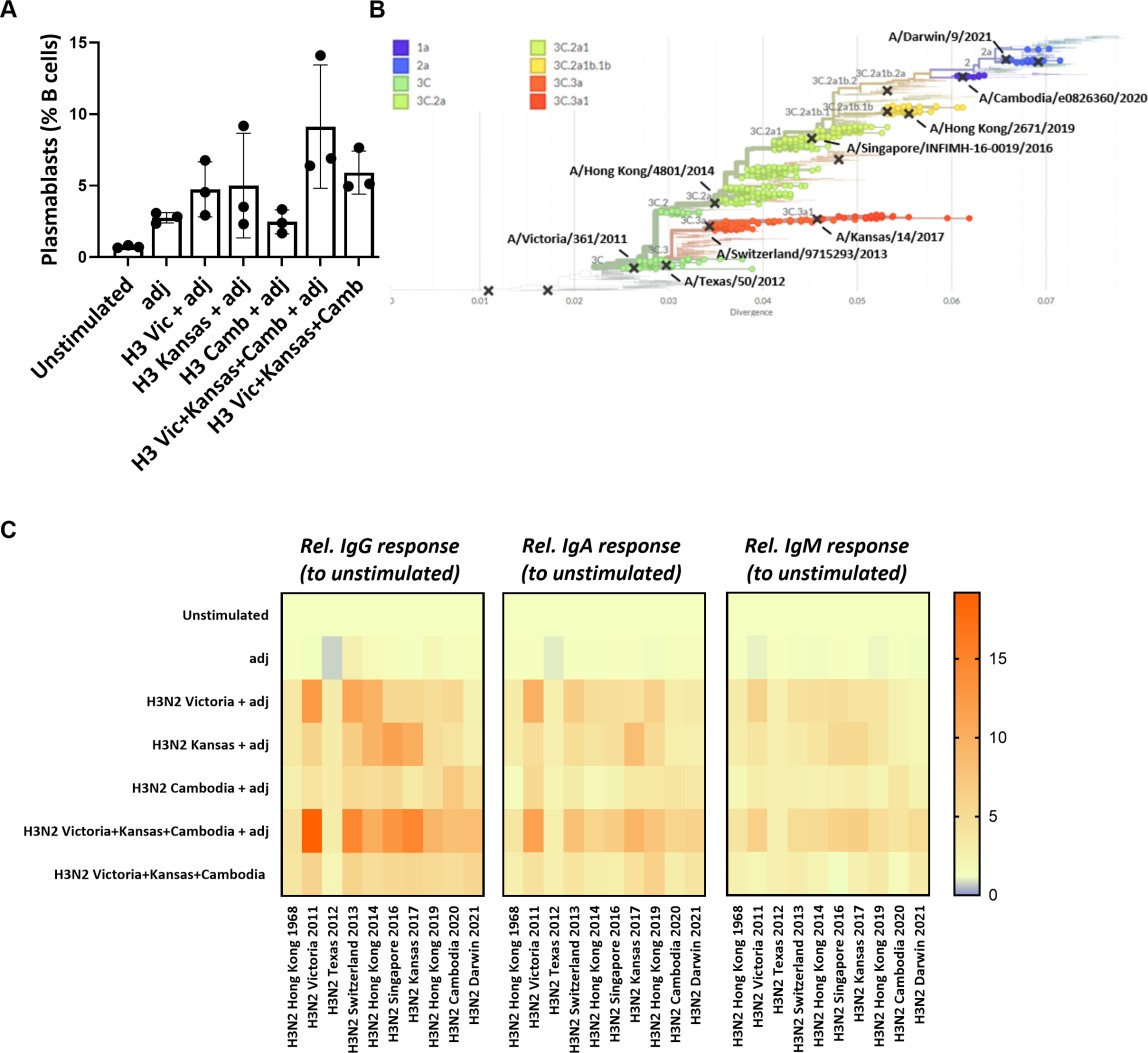
Multivalent vs monovalent influenza HA antigen *in vitro* immunization. **(A)** Plasmablast differentiation after 5-day stimulation was determined by flow cytometry (n=3). **(B)** Phylogenetic tree showing the antigenic relatedness of the influenza H3N2 strains included in vaccines between 2013/2014 and 2022/2023. The graph was generated using *nextrain.org* (49, 50). **(C)** Multiplexed, Luminex-based influenza strain-specific antibody measurements against the HA protein of ten influenza H3N2 strains. The y-axis indicates the antigenic stimulation condition used for *in vitro* immunization in the 3D perfusion bioreactor; the x-axis indicates the magnitude (fold increase) of the H3N2 strain-specific IgG/IgA/IgM responses normalized to unstimulated condition upon 5-day stimulation (n=3). Values were compared using Friedman test with Dunn’s correction. adj = adjuvant (=LPS).

Given the high mutation rate of influenza viruses, immune escape from pre-existing antibodies poses a significant challenge in influenza vaccine design. We next tested whether our *ex vivo* lymphoid culture system could be employed to improve vaccine antigen selection. First, we used the HA proteins from three different influenza H3N2 strains (Victoria 2011, Kansas 2017, Cambodia 2020) either alone (‘monovalent’ immunization) or in combination (‘multivalent’ immunization) to test for antigenic interference. We observed that plasmablast differentiation was not lower after the H3N2 multivalent *in vitro* immunization (9.1 ± 4.3%) compared to the monovalent conditions (Victoria 2011: 4.7 ± 1.9%, Kansas 2017: 5.0 ± 3.7%; Cambodia 2020: 2.5 ± 0.8%) (**Figure 4A**). Next, we measured the IgG, IgA, and IgM responses against the influenza H3N2 strains used for the *in vitro* immunization and assessed the cross-reactivity against historical or future influenza H3N2 strains (**Figure 4B**). This cross-reactivity analysis also included the antigenically very distant H3N2 Hong Kong/1/1968 pandemic strain as a comparator. Despite using the same total HA concentration, we found more robust and broader IgG, IgA, and IgM responses upon multivalent compared to the monovalent immunization. In particular, Victoria 2011-specific, Kansas 2017-specific, and Cambodia 2020-specific IgG responses were higher after multivalent stimulation than the corresponding monovalent immunization (fold increase relative to unstimulated: 19.1 vs. 12.9; 14.7 vs. 10.2; 8.0 vs. 7.1, respectively). Similar patterns were observed for the strain-specific IgA (11.4 vs. 9.9; 9.4 vs. 8.4; 5.8 vs. 4.1) and IgM (6.4 vs. 5.9; 6.5 vs. 5.4; 4.1 vs. 3.8). Notably, the multivalent condition was also associated with humoral responses to the newly emerged H3N2 strain Darwin 2021 that included specific IgG (8.4-fold increase), IgA (6.1-fold increase), and IgM (4.9-fold increase). Contrarily, we found no Darwin-specific responses upon the monovalent stimulations, except for IgG (4.9-fold increase) after stimulation with Cambodia 2020 (**Figure 4C**; **Supplementary Figure S7**). Thus, multivalent immunization showed no signs of antigenic interference but increased the breadth of the humoral response and elicited a response to an emerging antigen strain not included in the antigens used for immunization.

### *In vitro* immunization with three phylogenetically distant H3N2 strains generates the broadest immune response in tonsils

**Figure 5:**
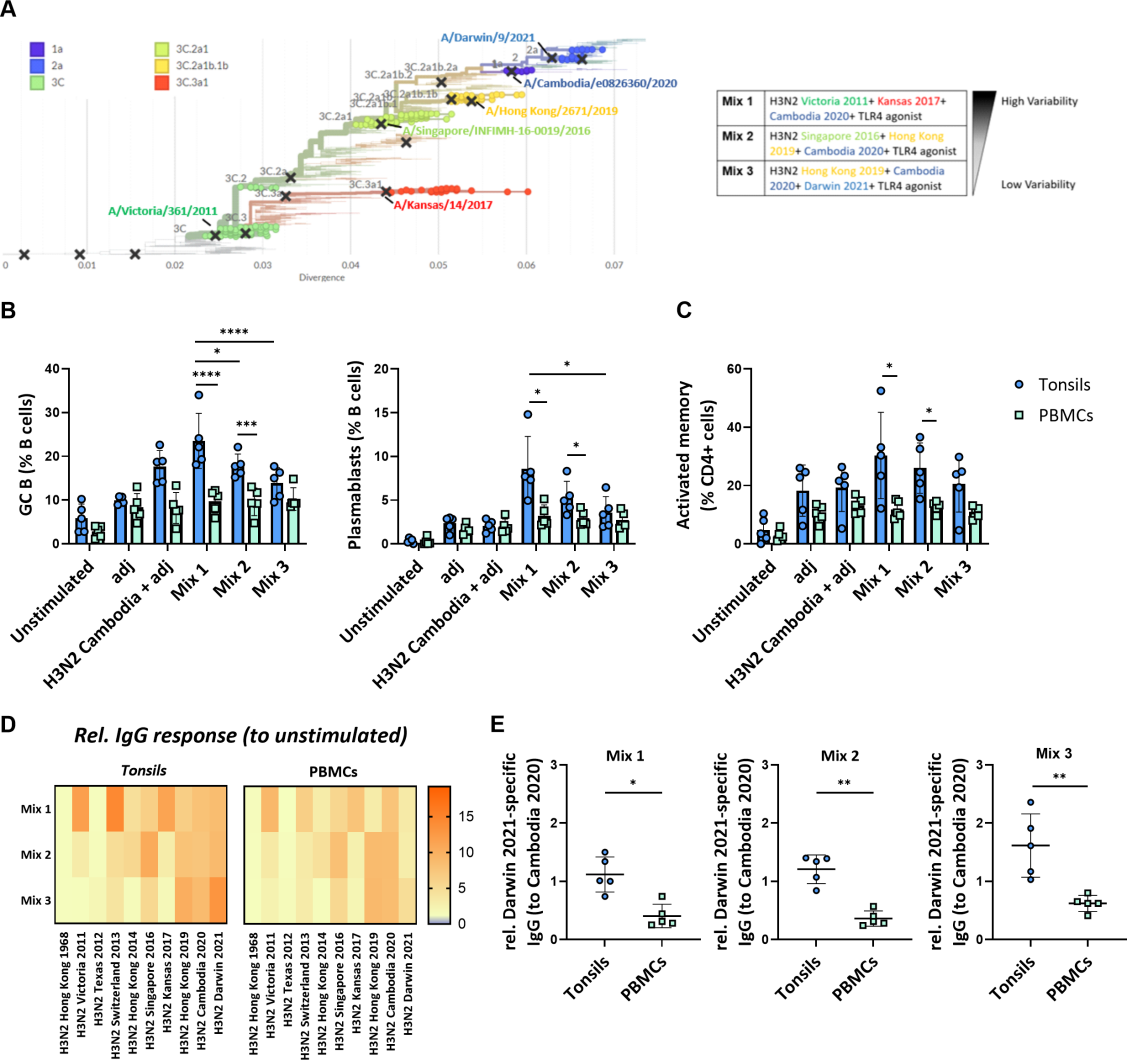
Impact of the antigenic diversification in multivalent H3N2 *in vitro* immunizations. **(A)** Phylogenetic tree highlighting the specific clades and H3N2 strains selected for multivalent HA antigen stimulations. The indicated distinct multivalent stimulation conditions (mix 1-3) were tested. **(B)** Effect of the different H3N2 antigenic compositions on the *in vitro* immunization-induced GC B cells and plasmablast differentiation in tonsil and PBMC samples (n=5). **(C)** Frequency of activated memory CD4^+^ T cells induced by *in vitro* multivalent stimulation conditions based on the expression of HLA-DR and CD45RA (n=5). adj = adjuvant (=LPS) **(D)** Influenza strain-specific antibodies in the supernatant of the cultured tonsils and PBMC upon 5-day stimulation were quantified by multiplexed Luminex assay. The y-axis shows the stimulation condition, while the x-axis shows the relative specific IgG responses against ten H3N2 strains. Luminex-derived median immunofluorescent (MFI) data was normalized to the MFI of the unstimulated condition; **(E)** H3N2 Darwin 2021-specific IgG normalized to H3N2 Cambodia 2020 response in tonsils vs PBMC (n=5). Two-way ANOVA with Turkey’s *post hoc* test and Mann-Whitney test were used to compare several or two groups as appropriate. *p<0.005, **p<0.001, ***p<0.0001, ****p<0.0001.

We next examined how the antigenic distance between strains in different multivalent conditions affects the immune response in our *in vitro* system. We stimulated the perfusion-cultured tonsils with three differently composed H3N2 multivalent conditions. Specifically, we compared a condition consisting of three phylogenetically highly distant H3N2 strains (Mix 1: H3N2 Victoria 2011, H3N2 Kansas 2017, H3N2 Cambodia 2020), an intermediate condition with moderately distant strains (Mix 2: H3N2 Singapore 2016, H3N2 Hong Kong 2019, H3N2 Cambodia 2020), and a condition with three phylogenetically closely related H3N2 strains (Mix3: H3N2 Hong Kong 2019, H3N2 Cambodia 2020, H3N2 Darwin 2021) (**Figure 5A**; **Supplementary Figure S8**). In parallel, we compared the immune response induced in tonsils from the different multivalent conditions to stimulations performed on peripheral blood immune cells from the same donors. After 5-days, we found significantly higher frequencies of GC B cells and plasmablasts in the tonsil compared to PBMC cultures (**Figure 5B**). The antigen mix with the highest antigenic variability induced the most potent tonsil B cell response compared to stimulated PBMC (GC B cells 23.5 ± 6.3 vs. 9.7 ± 2.7%, p<0.0001; plasmablasts 8.6 ± 3.7 vs. 3.2 ± 1.3%, p=0.027) (**Figure 5B**). Beyond these differences in the B cell compartment, we also observed that the fraction of activated memory CD4^+^ T cells was significantly higher upon stimulation with mix 1 in tonsils compared to PBMC (30.3 ± 14.8 vs. 11.8 ± 2.9%, p=0.047) (**Figure 5C**). The other antigenic conditions showed similar trends. We also observed activation in CD8^+^ T cells in both tissues and PBMC (**Supplementary Figure S9**).

To assess the functional consequences of the antigenic composition on the humoral immune response, we determined the cross-reactivity of the IgG antibodies produced upon stimulation with the different multivalent conditions in both tonsils and PBMC. We observed a broader response upon immunization with the three phylogenetically distant H3N2 strains compared to the stimulation conditions with more related strains. The IgG response in tonsil cultures was higher than in PBMC cultures from the same donors (**Figure 5D**). Interestingly, antibodies against the most recent H3N2 strain, Darwin 2021, were only elicited in immunized tonsils but not PBMC (relative response to Cambodia 2020: 1.12 vs. 0.41, p=0.0159 for mix 1; 1.21 vs. 0.36, p=0.0079 for mix 2; 1.62 vs. 0.60, p=0.0079 for mix 3 that contained Darwin) (**Figure 5E**).

The fact that PBMC and tonsils respond differently to vaccine combinations shows the potential value of 3D models like perfusion-cultured tonsils in studying influenza responses. The *ex vivo* perfusion-based tonsil model was superior to stimulations of peripheral blood cells and sensitive enough to reveal different responses towards three phylogenetically H3N2 strains with different antigenic distances. Furthermore, the Darwin 2021-specific antibody response, even in the absence of the respective antigen in mix 1 and 2, may be explained by either cross-reactive or *de novo* antibodies. Therefore, the generation of *de novo* responses in our *in vitro* model needed to be investigated.

### Perfusion-cultured tonsils support *de novo* immune responses

**Figure 6:**
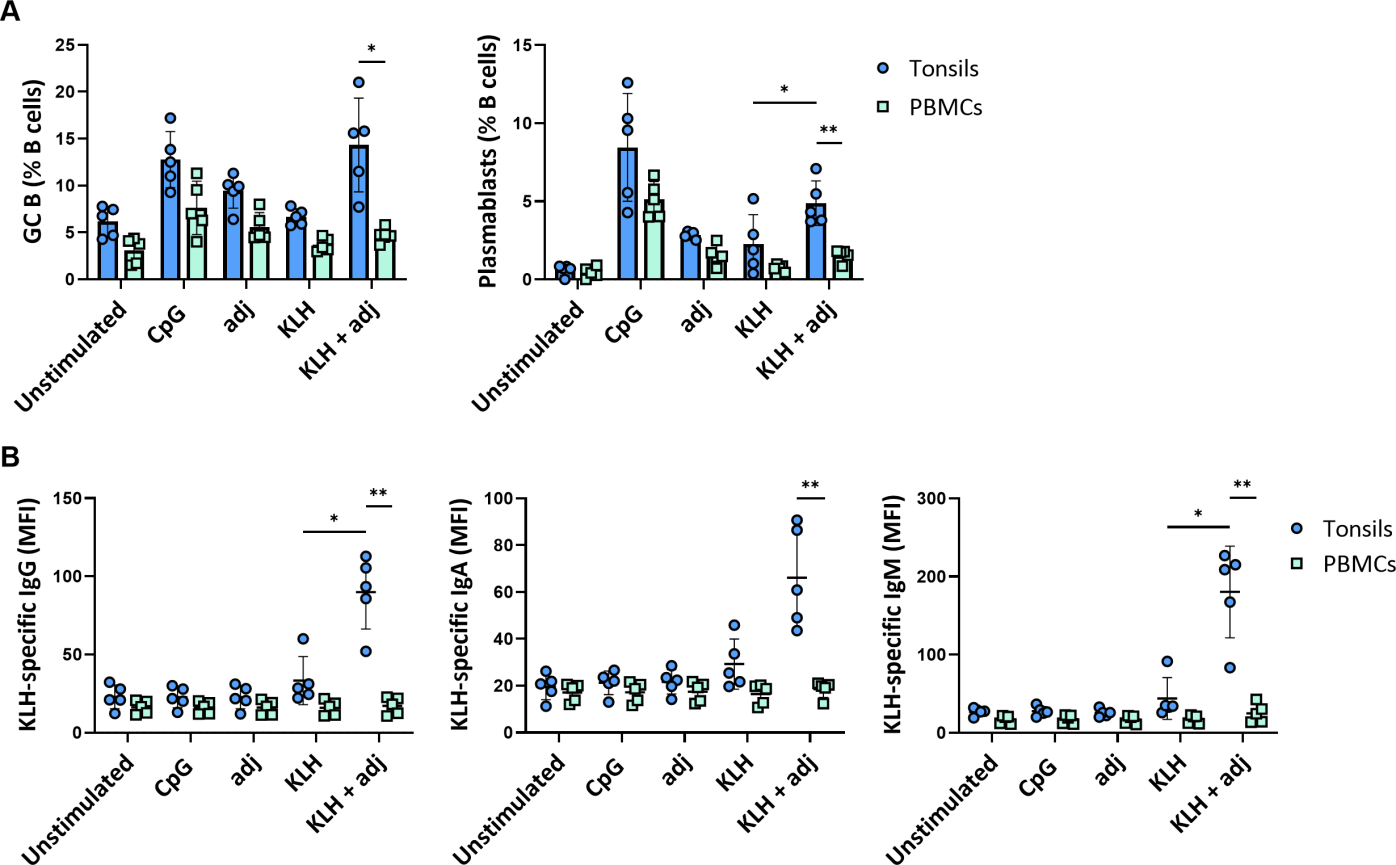
*De novo* B cell responses to a neo-antigen. **(A)** GC B cell and plasmablast frequencies at 5 days following stimulation with KLH of tonsils and PBMC (n=5). **(B)** KLH-specific IgG, IgA, and IgM responses in tonsil and PBMC samples (n=5). Statistical significance was determined using two-way ANOVA with Turkey’s *post hoc* test. KLH = keyhole limpet hemocyanin; adj = adjuvant (=LPS). *p<0.005, **p<0.001.

The immune response after *in vitro* stimulation of immune cells may be dominated by recall responses of pre-existing antigen-specific cells. Whether such systems generate *de novo* responses upon antigen stimulation is critical for their potential use in vaccine development. Consequently, we investigated the potential of our *ex vivo* lymphoid culture system by stimulating perfusion-cultured tonsils with a bonafide neo-antigen in the presence or absence of the same adjuvant as with the influenza proteins. In particular, we compared Keyhole Limpet Haemocyanin (KLH) protein stimulations of tonsil tissue vs PBMC. Strikingly, GC B cell and plasmablast frequencies were significantly increased after stimulation with KLH combined with the adjuvant compared to the unstimulated condition in tonsils (GC B cells: 6.2 ± 1.6% vs. 14.3 ± 4.9%; plasmablasts: 0.6 ± 0.3% vs. 4.8 ± 1.4%, respectively) but not in PBMC (GC B cells: 3.1 ± 1.4 vs. 4.7 ± 0.8; plasmablasts: 0.5 ± 0.3 vs. 1.5 ± 0.4, respectively) (**Figure 6A**). Plasmablast differentiation resulted in KLH-specific antibody secretion in perfusion-cultured tonsils. We observed significant production of specific IgM (MFI 26.9 vs. 180) and, to a lesser extent, of IgG (MFI 22.9 vs. 89.9) and IgA (MFI 19.5 vs. 66.1), consistent with a *de novo* immune response. Contrarily, we did not find an increase in KLH-specific antibodies in cultures of PBMC upon stimulation with KLH (**Figure 6B**).

Thus, tonsils cultured in 3D perfusion bioreactors allow us to generate *de novo* responses and predict differential responses to complex vaccine design strategies.

## Discussion

Vaccination is an effective preventive measure against infection. Pathogens with relatively high mutation rates, including influenza or SARS-CoV2, continuously generate novel antigenic variants with the potential to escape recognition by vaccination-induced antibodies. Designing vaccines that induce broadly neutralizing antibodies to highly mutating viruses is exceptionally challenging and affected by patient heterogeneity and the individual pre-existing immune state. For example, the first exposure to an antigen shapes the lifelong immunity against related antigens (8). Moreover, the lack of screening platforms that account for the complexity of human immune responses slows the development of vaccines with broader antigenic coverage and efficacy, especially for highly variable viruses. Generating new experimental approaches that mirror features of human immunity would represent a significant advantage in vaccine design. Here, we developed an *in vitro* vaccination model based on the *ex vivo* culture of tonsil explants in 3D perfusion bioreactors. First, we demonstrated that using a bioreactor to perfuse the tissue with medium supports the maintenance of functional tonsil tissues better than a static culture system. We substantiated this by showing higher viability, cellular fitness, and cell density of perfusion-cultured tonsils over time, as well as a more robust humoral immune response to antigenic stimulation. Secondly, we showed that perfusion-cultured tonsils reveal different strengths and cross-reactivity of antibody responses following immunization with premanufactured vaccines but also using recombinant protein antigens combined with an adjuvant. This allowed us to study the generation of cross-reactive immunity following different multivalent antigen combinations in *in vitro* immunization. Our data indicated that using three phylogenetically distant H3N2 influenza strains induced a broader immune response while including similar strains enforced the response to the most recent strain. Finally, this *in vitro* model of a secondary lymphoid organ -but not PBMC- supported the generation of *de novo* adaptive immune responses to a classic neo-antigen (i.e., KHL).

Different secondary lymphoid organs could be used for *in vitro* immunization models. We took advantage of tonsils since they are available from routine tonsillectomies. A downside of tonsil samples is that they may not be considered entirely normal tissue since the most frequent indication for tonsillectomy is recurrent tonsillitis. One way to circumvent this would be using tonsillectomies performed for sleep apnea syndrome. In our clinic, this surgery is rarely done. Despite this minor concern, tonsils are the most accessible model for studying adaptive immune response compared to the other secondary lymphatic organs, such as lymph nodes or spleen, which are more challenging to obtain or are too small for experimental purposes. As a limitation, each human specimen is characterized by a unique clonal cell composition and shaped by the individual immunological experience, increasing the complexity of data interpretation. Moreover, age of the patient and the general condition of the tonsil may affect the outcome of *in vitro* immunization, which should be addressed in larger studies. Since we analyzed a relatively small sample size, we cannot exclude higher variability in the outcomes if a more prominent number of tonsils were tested per experimental conditions.

Another critical aspect of an *in vitro* immunization model is the culture condition. We describe that 3D perfusion bioreactors better maintained tissue viability, cell density, and cellular functionality over time than the conventional static cultures. In bioreactors, constant perfusion of the culture medium through the tissues reduces the formation of chemical gradients (oxygen, CO_2_, antigens, nutrients, metabolic wastes), preventing cell death and mimicking better the *in vivo* situation (38–40). However, the viability of tonsils started to decrease from day 5 to 14, similar to other *in vitro* cell-based models (41–43). Therefore, further efforts should be focused on identifying factors that could promote tissue survival over a more extended culture period. Moreover, the assessment of cell viability and metabolic activity could then include additional assays that characterize various aspects of the cell condition and would be more cell-sparing, such as LDH measurement in the supernatants, use of a fluorescent reporter dye for mitochondrial activity, and measuring cellular glucose uptake. Moreover, further studies should elaborate on how adapted perfusion protocols may impact viability in central vs. peripheral parts of the cultured tonsil tissue. The reduced cellular viability precluded meaningfully studying later time points. Therefore, it is unclear to which extent GC-derived plasma and memory cells were produced in our system. Extra-follicular B cell responses potentially dominated the observed immune responses due to the shorter assay time. However, the fact that GC B cells were robustly induced suggests that at least some GC response was occurring. Finally, another major improvement of the bioreactor setup would be to reduce the size of each perfusion chamber to increment the replicates (44).

Several *in vitro* models have been developed in recent years to study antigen-specific immune responses. Most of these systems used murine or human immune cells combined with scaffolds to recapitulate some aspects of lymphoid tissues (22–26). One study illustrated that tonsil cells -cultured in bioreactors- can generate antigen-specific antibody responses (28). Others used cryopreserved tonsil cells cultured in plates as an *in vitro* immunization system. In this work, tonsil organoids could respond to both recall and neo antigens and support key GC features, including antigen-specific somatic hypermutation, affinity maturation, and class switching (27, 29). Similar to our *in vitro* model, they induced immune responses against neoantigens and previously encountered antigens with a significant increase in GC B cells. However, our analyses were limited to the early events of the immune response, and, thus, we could not investigate GC events such as somatic hypermutation by B cell receptor sequencing. Despite the ability to induce adaptive immunity, models using tonsil single-cell suspensions do not fully replicate the cellular organization of lymphoid tissue. Moreover, the composition and distribution of specific immune cell subsets may have been altered by cryopreserving the cells before use. Instead, we used tonsil explants in their native 3D structure that includes stromal cells. Here we focused on B cell immunity, but further studies should include an extended characterization of these cells using stromal cell-specific markers. Notably, the tissues maintained the immunological architecture of a secondary lymphoid organ over the culture period. Follicles contained a high frequency of cells positive for CD20 and Ki67, consistent with B cell proliferation and CD21, indicating FDCs within lymphoid follicles. Notably, CD21 is also expressed by a small fraction of B cells; thus, we cannot exclude that some CD21-positive cells were B cells. Notably, we observed no strong increase in Ki67, which could have been due to the timing of sampling or the stimulation conditions. Further studies should include additional cell proliferation markers and assess Ki67 in flow cytometry. In summary, a 3D system may better reflect the *in vivo* situation than systems using cryopreserved single-cell suspensions, but direct comparisons would be needed to define the assay-specific advantages and disadvantages of these approaches when using identical experimental conditions.

Perfusion-cultured tonsils provide a valuable platform for characterizing immune responses to influenza viruses. In exploratory work, we identified that combining the recombinant protein antigens with a TLR4 agonist as the adjuvant enabled more robust adaptive immune responses. Specifically, the TLR4-induced immune stimulation was better than TLR7 stimulation with CpG, possibly due to better activation of T helper cells via cytokines released from antigen-presenting cells in our *in vitro* model. Notably, TLR4 agonists are used in commercial vaccines such as the adjuvanted recombinant shingles vaccine. An interesting aspect is the increase of GC B cell subset upon stimulation with all three H3N2 multivalent conditions, consistent with successful induction of GC responses. Conversely, we have not observed a GC B cell increase when using autologous PBMC and the same antigen stimulations, suggesting that the differences in the cellular composition between tonsils and PBMC and the structural organization of the tonsils matter. Notably, memory B cells and T follicular helper cells (Tfh) are more frequent in the tonsils compared to the peripheral blood immune cell compartment, potentially promoting better adaptive immune responses (45). Future studies should investigate the role of T cell subsets, particularly Tfh, in the *in vitro* vaccination model as well as the generation of antigen-specific T cells upon stimulation. Differences in the B cell activation were associated with a lower IgG production in cultures using PBMC compared to tonsils, but the overall cross-reactivity pattern of the antibodies was similar. However, we cannot exclude that the smaller proportion of B cells in PBMC may have also affected the results. Moreover, the broader coverage of the multivalent immunization with three phylogenetically distant H3N2 influenza strains may rely on the induction of several repertoires of cross-neutralizing antibodies targeting unique common epitopes, expanding the immune response. Characterization of these epitopes will be of interest, especially for developing a universal influenza vaccine. Affinity measurements could also provide helpful information regarding the quality of the induced humoral response, but studies with more extended time points are needed. Another important consideration is that tonsils incorporate pre-existing responses, particularly critical in influenza (7, 46). Pre-existing memory B cells were likely boosted upon *in vitro* tonsil immunization and may have contributed substantially to the observed antibody response. However, beyond recall responses, we have shown that our *in vitro* vaccination system -but not PBMC- could induce *de novo* responses. Whether *de novo* B cell responses depend on the spatial immune cell organization in the tissue, the cellular composition or the presence of stromal cells needs to be further defined. Interestingly, previous studies have demonstrated that KLH can promote maturation of dendritic cells (DCs) via the mannose receptor (47). Considering the higher frequency of DC in tonsils compared to the peripheral blood, further efforts should be focused on identifying the role of DCs in the response upon KLH stimulation in tonsil (48). In addition, testing of other neo antigens should be included in future works. Remarkably, this model can support immune responses against different antigens and antigen combinations, underpinning its applicability in vaccine candidates or antigen screening.

Altogether, the presented human *in vitro* model of secondary lymphoid organ is a promising platform for testing and comparing vaccine candidates to new and previously encountered antigens. Future applications of this system can also include the analysis of the effects of immunomodulatory drugs, and adjuvants, and studying affinity maturation in germinal centers depending on antigen combinations.

## Data Availability

The raw data supporting the conclusions of this article will be made available by the authors, without undue reservation.
