# Supplementary material for: Tonsil explants as a human in vitro model to study vaccine responses
Source: Front Immunol. 2024 Sep 17;15:1425455. doi: 10.3389/fimmu.2024.1425455 (PMC11442277; doi:10.3389/fimmu.2024.1425455)
Supplement: Supplementary file 1 [file DataSheet1.docx]

**Supplementary Information**

Tonsil explants as a human *in vitro* model to study vaccine responses.

Bonaiti E. et al.

Supplementary Fig. 1. Experimental design of the study.

Supplementary Fig. 2. T cell gating strategy for flow cytometry.

Supplementary Fig. 3. Maintenance of cell density in perfusion-cultured tonsils.

Supplementary Fig. 4. Gating strategy for B cell subset immunophenotyping.

Supplementary Fig. 5. Total IgG in response to influenza vaccine or CpG stimulations in tonsils.

Supplementary Fig. 6. Cytokine analysis in culture supernatants of influenza vaccine or CpG stimulated perfusion-cultured tonsils.

Supplementary Fig. 7. Influenza strain-specific antibody production following H3N2 monovalent or multivalent *in vitro* stimulations.

Supplementary Fig. 8. HA variability of the H3N2 influenza strains in the different multivalent conditions.

Supplementary Fig. 9. T cell activation upon *in vitro* stimulation with different H3N2 mixes in tonsil vs. PBMC.


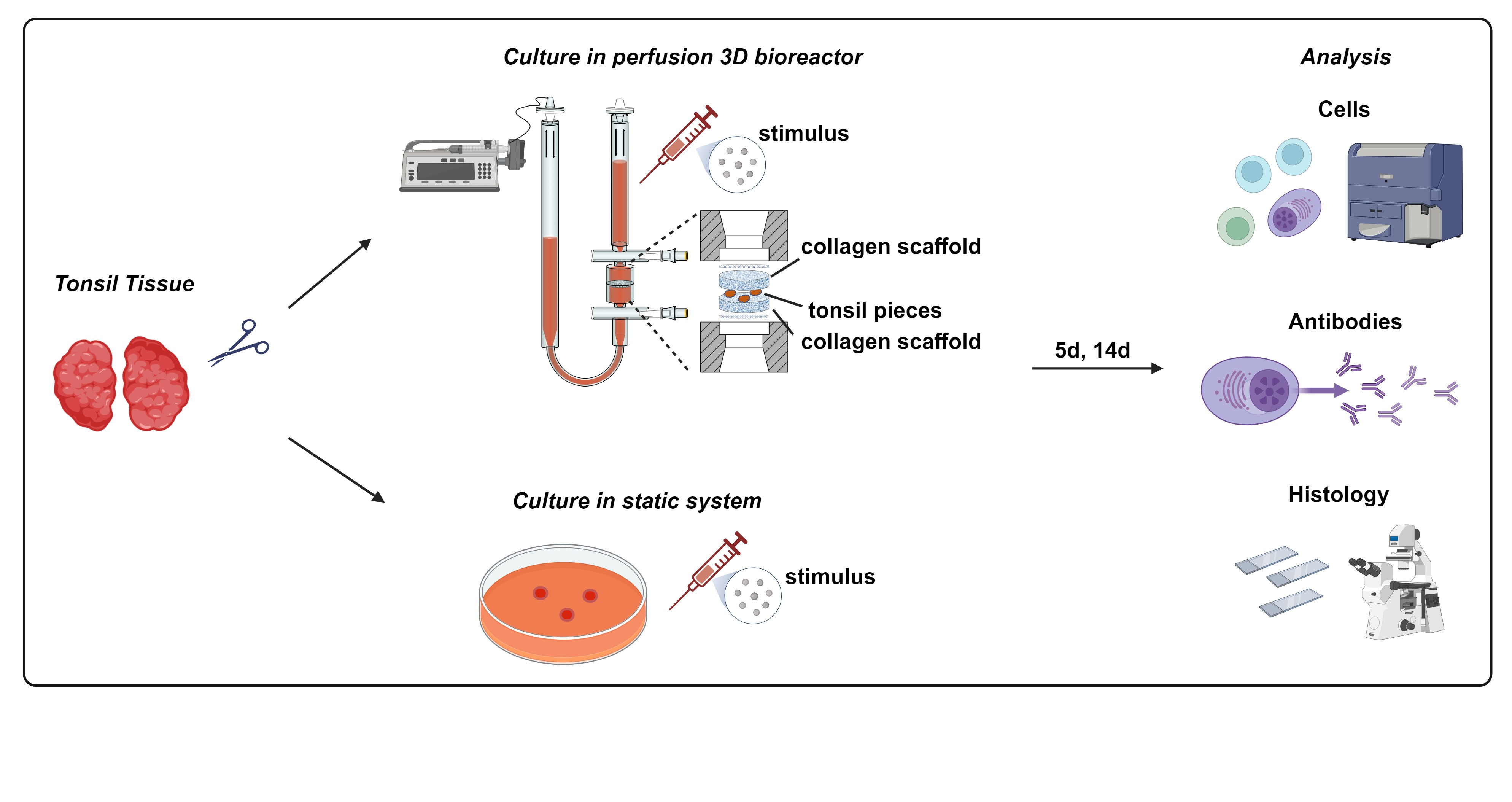


**Supplementary Fig. 1. Experimental design of the study.** Tonsils were cultured in perfusion 3D bioreactor vs. static system in unstimulated and stimulated conditions for 5 days and 14 days. Immunophenotype, tissue morphology and architecture, and antibody production were assessed at the end of the culture period. Create with Biorender.com.

**
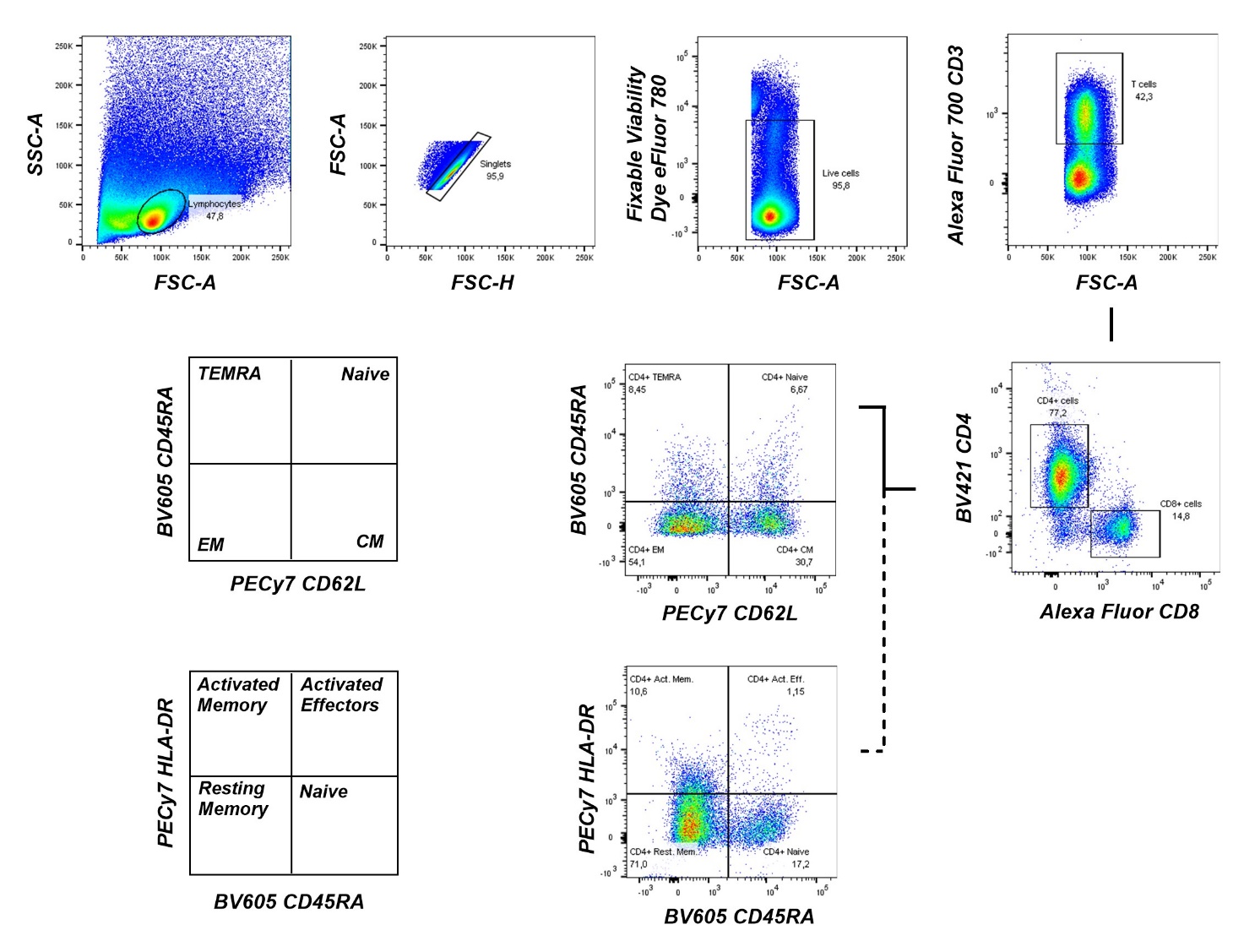
**

**Supplementary Fig. 2. Gating strategy for T cells.** Flow cytometry plots showing the gating strategy applied to determine T cell subsets and the activation status. CD3+ cells were gated on CD4+ and CD8+ cells. CD4+ cells were further phenotyped based on CD45RA and CD62L expression into naïve T cells, effector memory (EM), central memory (CM), and terminally differentiated effector memory (TEMRA) T cells. Using a different panel, activated memory, and effector CD4+ T cells were identified based on the marker HLA-DR and CD45RA. The same strategy was applied to determine the different subsets among CD8+ cells.


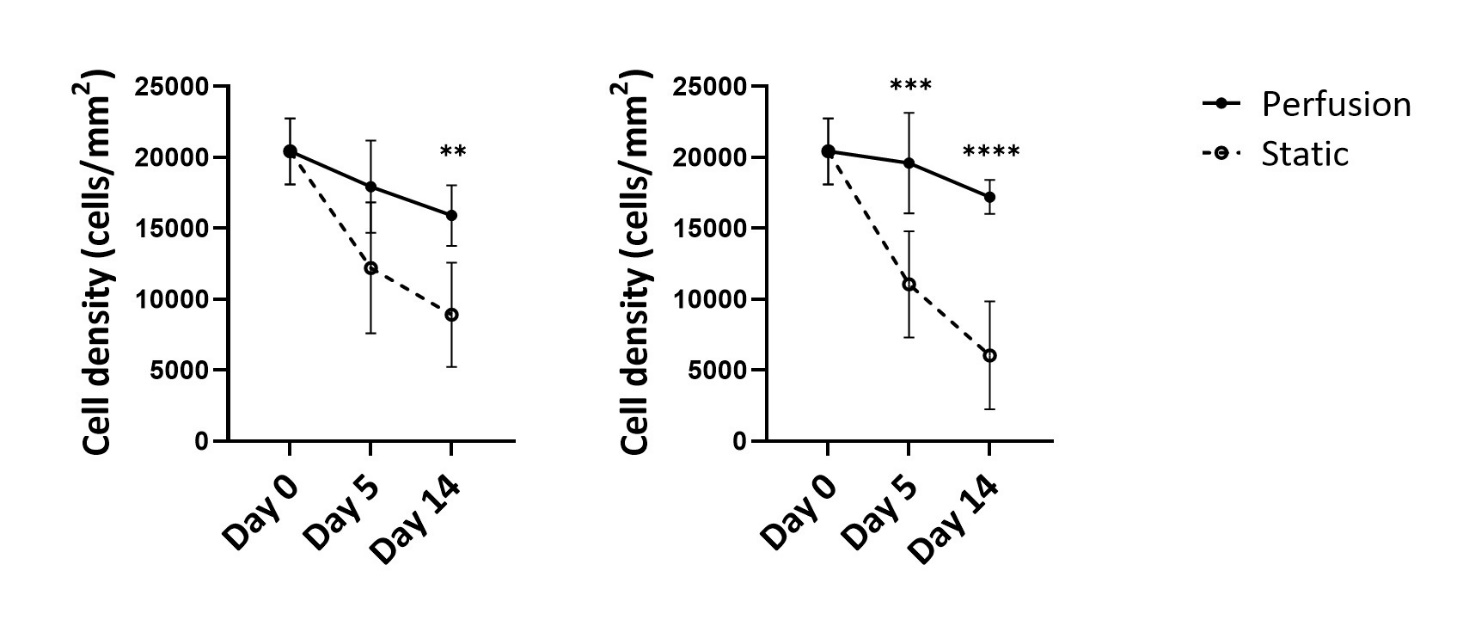
**Supplementary Fig. 3. Maintenance of cell density in perfusion-cultured tonsils.** Quantification of cell density as total cell number versus tissue area at day 0, 5 and 14 in unstimulated and influenza vaccine stimulated conditions (n=7). Quantification was done using QuPath software, version 0.3.2. Statistical significance between groups was determined using two-way ANOVA with Bonferroni’s post hoc test. **p<0.001, ***p<0.0001, ****p<0.0001.


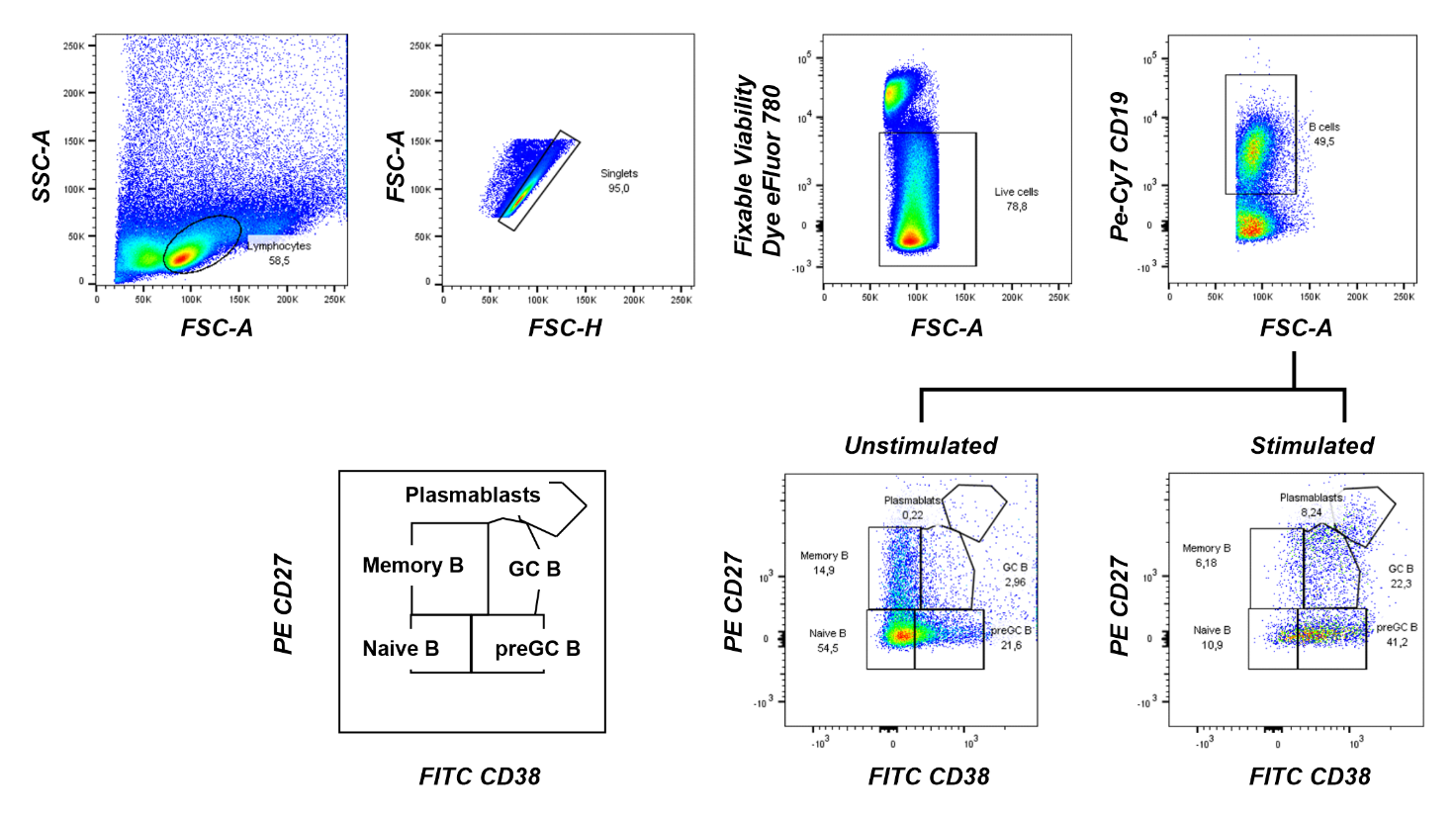


**Supplementary Fig. 4. Gating strategy for B cell subset immunophenotyping.** Flow cytometry plots show a representative example of the gating strategy applied to identify B cell subsets (unstimulated vs. stimulated condition is showed). CD19+ cells were phenotyped based on CD27 and CD38 expression into naïve B cells, memory B cells, pre-GC B cells, GC B cells and plasmablasts.


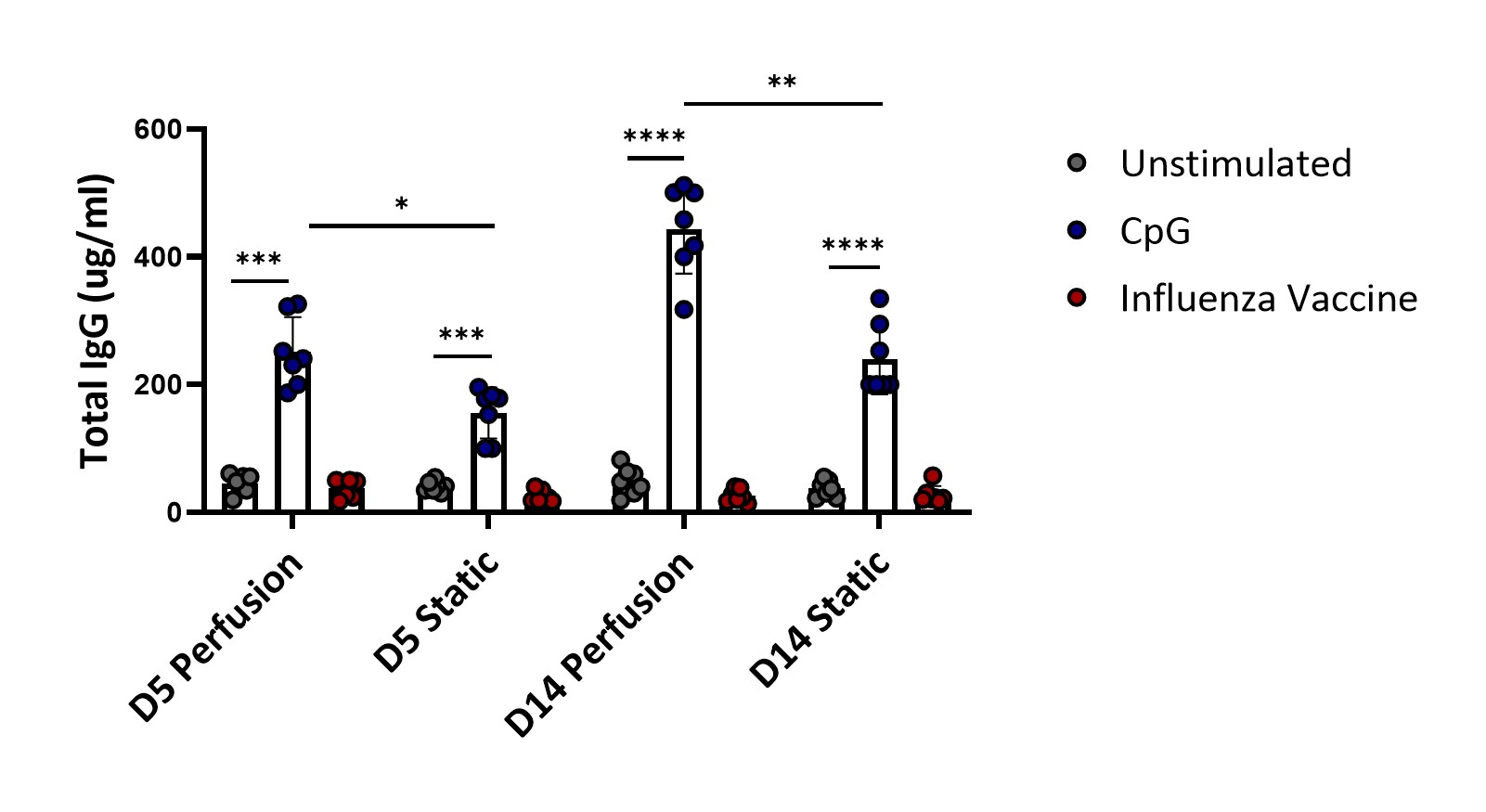
**Supplementary Fig. 5. Total IgG in response to influenza vaccine or CpG stimulations in tonsils.** Quantification of total IgG in the supernatants of tissue cultures (n=7) using bioreactors vs. static system at days 5 and 14. Two-way ANOVA with Turkey's post hoc test was used to determine statistical significance. *p<0.005, **p<0.001, ***p<0.0001, ****p<0.0001.


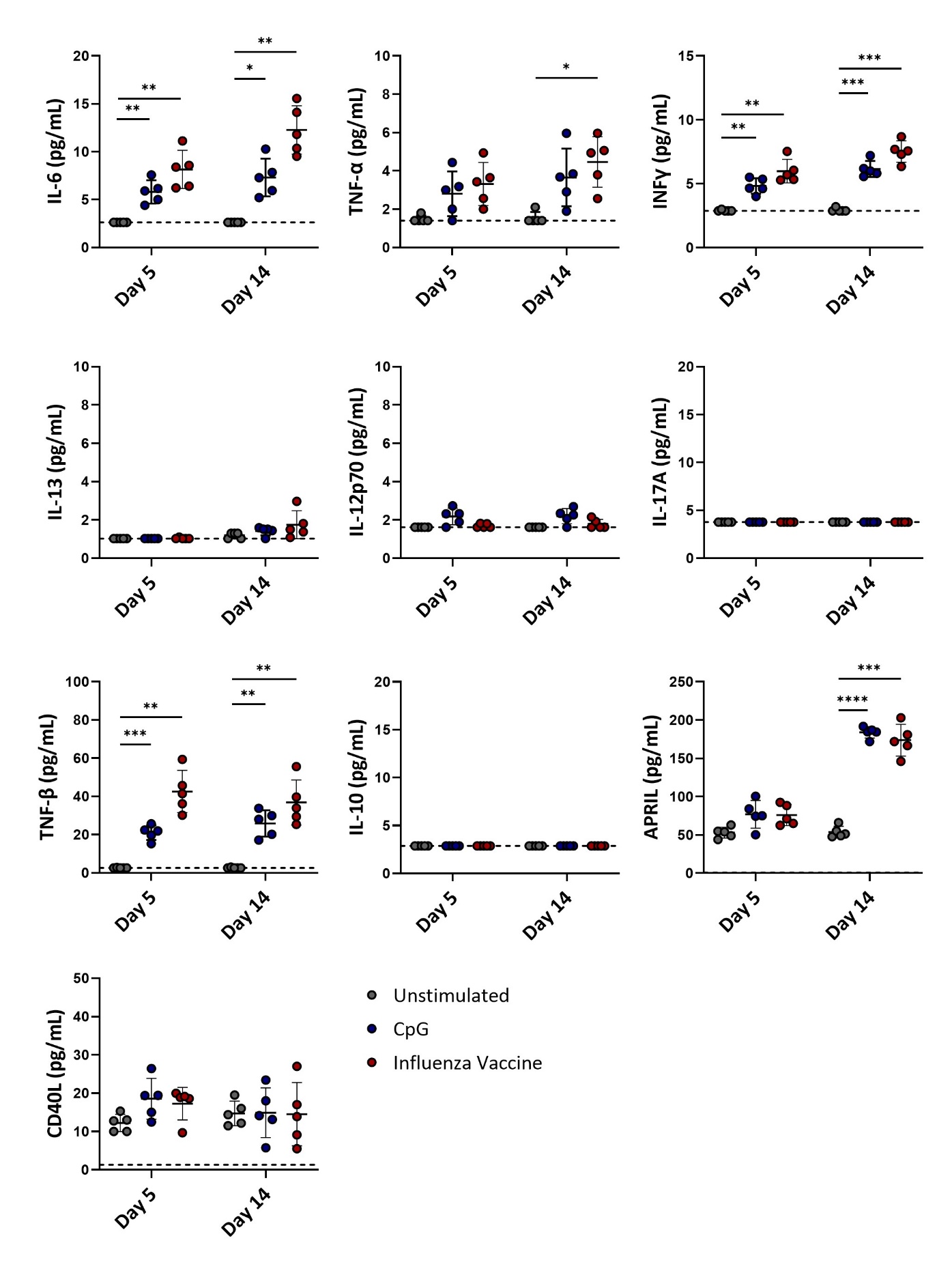


**Supplementary Fig. 6. Cell culture cytokine analysis in response to influenza vaccine or CpG stimulation from perfusion-cultured tonsils.** Quantification of cytokine levels in the supernatant of tonsils cultured in 3D perfusion bioreactors upon influenza vaccine or CpG stimulation (n=5). Statistical significance was determined by two-way ANOVA with Turkey's post hoc test. *p<0.005, **p<0.001, ***p<0.0001.


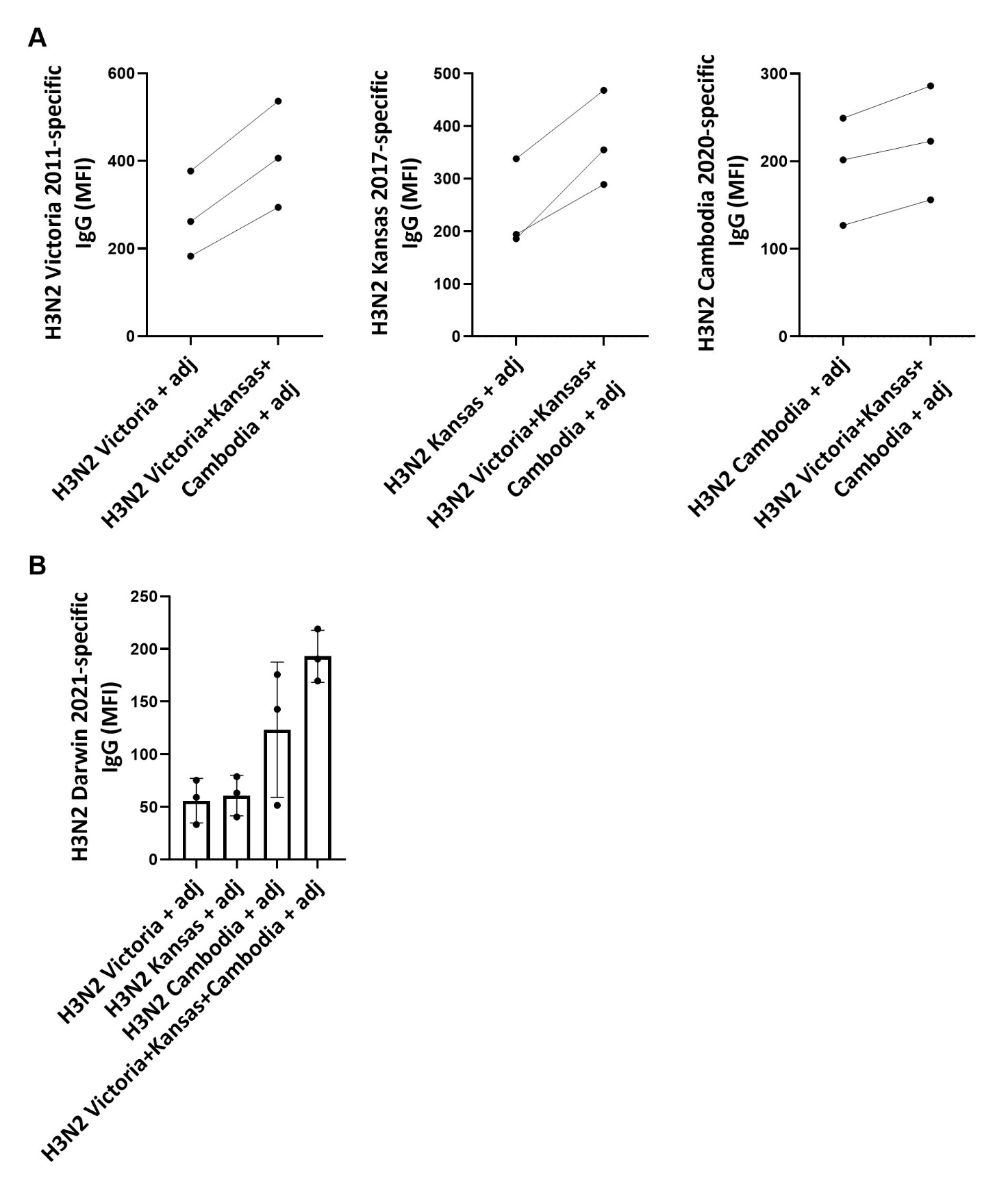
**Supplementary Fig. 7. Influenza strain-specific antibody production following H3N2 monovalent or multivalent *in vitro* stimulations. (A)** H3N2 Victoria 2011-, Kansas 2017-, Cambodia 2020-specific IgG (MFI) upon H3N2 multivalent *in vitro* immunization vs. the corresponding monovalent stimulation in tonsils (n=3). **(B)** H3N2 Darwin 2021-specific IgG responses in tonsils (n=3).


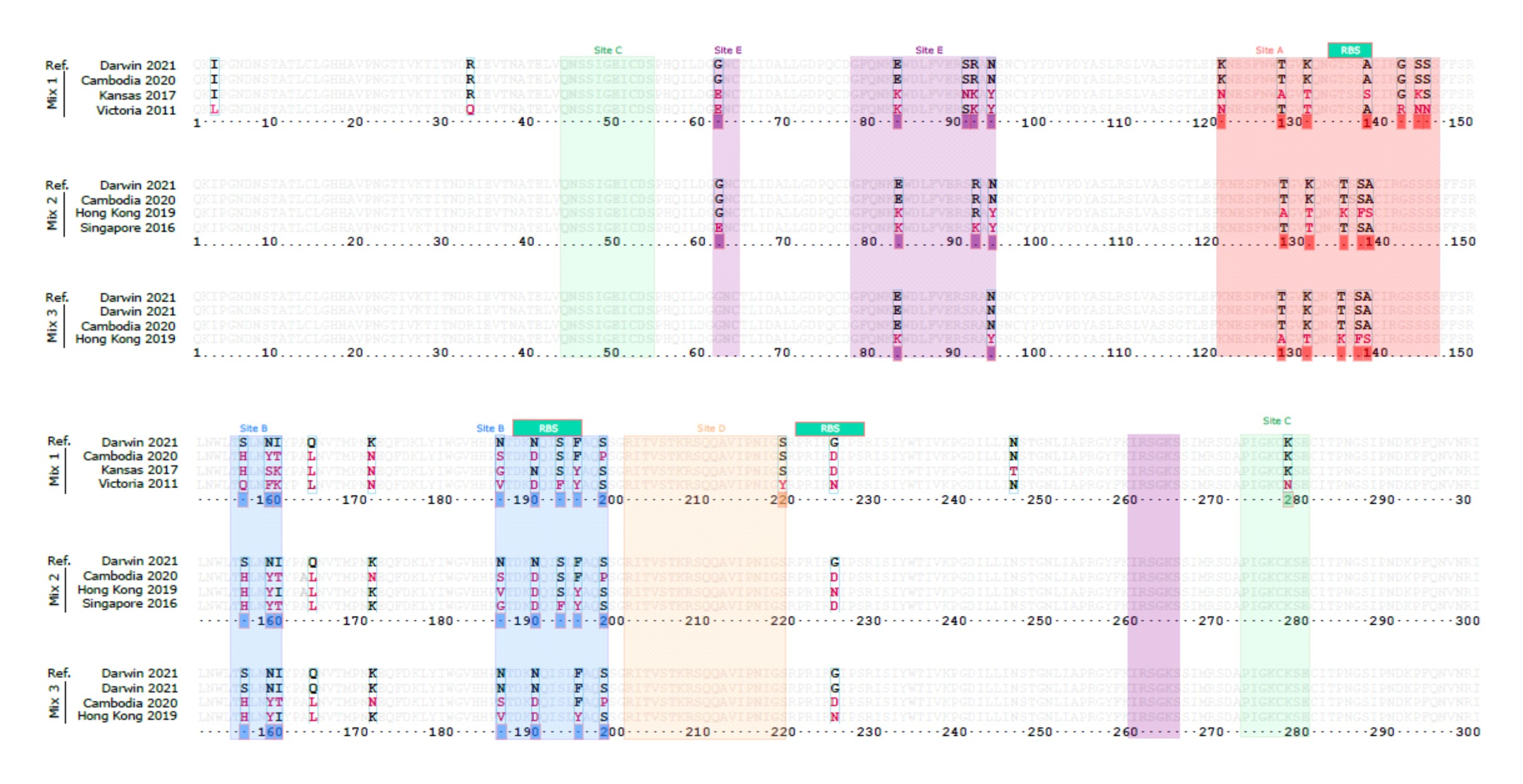


**Supplementary Fig. 8. HA variability of the H3N2 influenza strains in the different multivalent conditions.** Alignment of the amino acid sequences of HAs included in the mix 1, mix 2 and mix 3. The HA of H3N2 Darwin 2021 is used as reference. Substitutions included in the antigenic site A (red), B (blue), C (green), D (orange) and E (purple) are shown. Substitutions in the receptor binding site (RBS) are highlighted in brilliant green (1-3). Only the amino acids 1-300 including the antigenic sites and RBS are shown. Sequence alignment was performed in ClustalX 2.1.


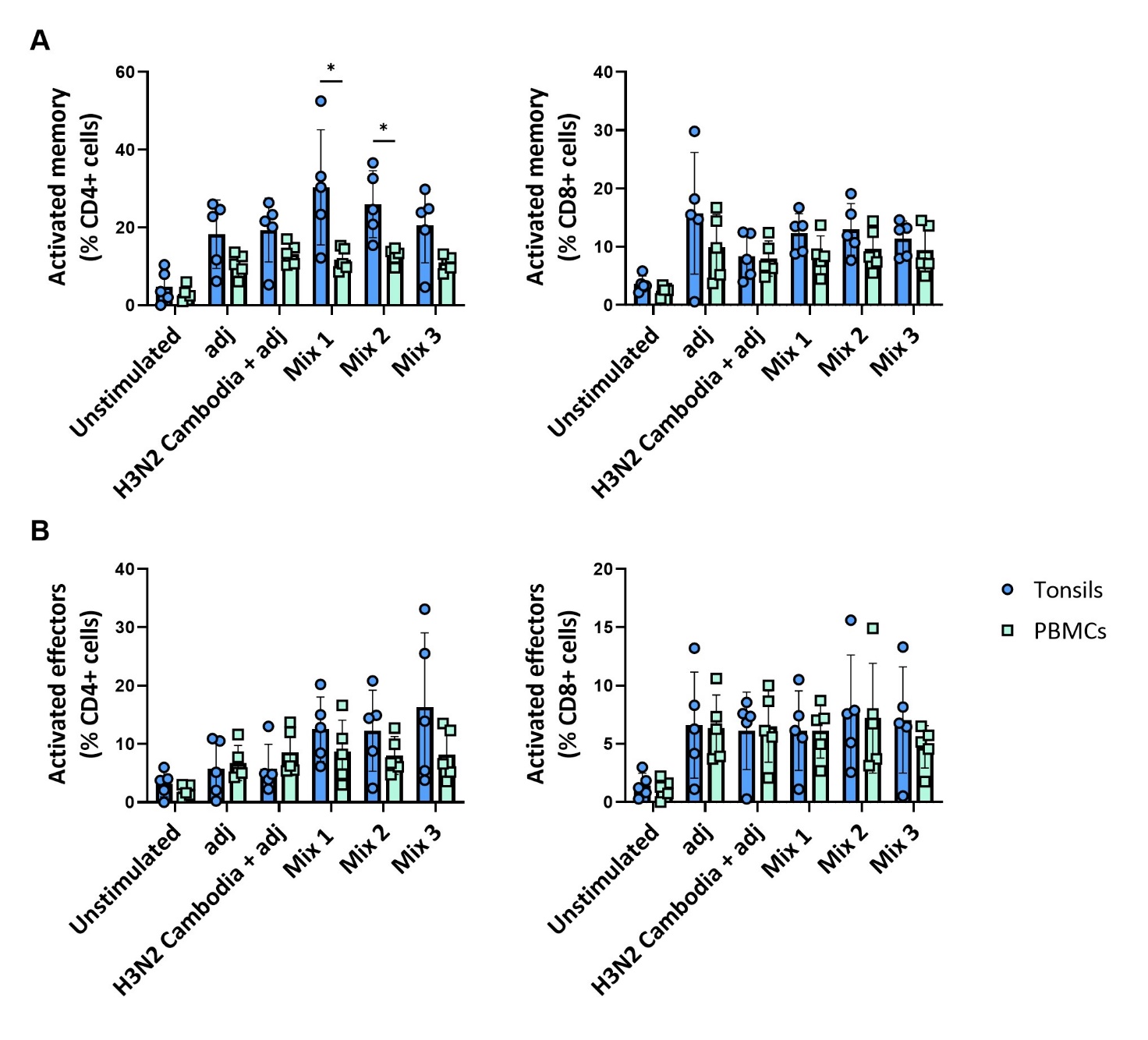
**Supplementary Fig. 9. T cell activation upon *in vitro* stimulation with different H3N2 mixes in tonsil vs. PBMC. (A)** Percentages of activated memory in CD4^+^ (also shown in Fig. 5) and CD8^+^ T subsets from tonsil vs. PBMC (n=5). **(B)** Frequencies of CD4^+^ and CD8^+^ activated effectors were determined by flow cytometry based on the markers HLA-DR and CD45RA in tonsil vs. PBMC (n=5). Values were compared by two-way ANOVA with Turkey’s post hoc test. *p<0.005.

**References**

1. Gerhard W, Yewdell J, Frankel ME, Webster R. Antigenic structure of influenza virus haemagglutinin defined by hybridoma antibodies. Nature. 1981;290(5808):713-7.

2. Lin YP, Xiong X, Wharton SA, Martin SR, Coombs PJ, Vachieri SG, et al. Evolution of the receptor binding properties of the influenza A(H3N2) hemagglutinin. Proc Natl Acad Sci U S A. 2012;109(52):21474-9.

3. Wiley DC, Wilson IA, Skehel JJ. Structural identification of the antibody-binding sites of Hong Kong influenza haemagglutinin and their involvement in antigenic variation. Nature. 1981;289(5796):373-8.
